# Maternal and infant predictors of infant mortality in California, 2007–2015

**DOI:** 10.1371/journal.pone.0236877

**Published:** 2020-08-06

**Authors:** Anura W. G. Ratnasiri, Satyan Lakshminrusimha, Ronald A. Dieckmann, Henry C. Lee, Jeffrey B. Gould, Steven S. Parry, Vivi N. Arief, Ian H. DeLacy, Ralph J. DiLibero, Kaye E. Basford

**Affiliations:** 1 Benefits Division, California Department of Health Care Services, Sacramento, California, United States of America; 2 School of Agriculture and Food Sciences, Faculty of Science, The University of Queensland, Brisbane, Queensland, Australia; 3 Department of Pediatrics, School of Medicine, University of California Davis, Sacramento, California, United States of America; 4 Pediatrics and Emergency Medicine, University of California San Francisco, San Francisco, California, United States of America; 5 Division of Neonatology, School of Medicine, Stanford University, Stanford, California, United States of America; 6 School of Biomedical Sciences, Faculty of Medicine, The University of Queensland, Brisbane, Queensland, Australia; University of New South Wales, AUSTRALIA

## Abstract

**Objective:**

To identify current maternal and infant predictors of infant mortality, including maternal sociodemographic and economic status, maternal perinatal smoking and obesity, mode of delivery, and infant birthweight and gestational age.

**Methods:**

This retrospective study analyzed data from the linked birth and infant death files (birth cohort) and live births from the Birth Statistical Master files (BSMF) in California compiled by the California Department of Public Health for 2007–2015. The birth cohort study comprised 4,503,197 singleton births including 19,301 infant deaths during the nine-year study period. A subpopulation to study fetal growth consisted of 4,448,300 birth cohort records including 13,891 infant deaths.

**Results:**

The infant mortality rate (IMR) for singleton births decreased linearly (p <0.001) from 4.68 in 2007 to 3.90 (per 1,000 live births) in 2015. However, significant disparities in IMR were uncovered in different population groups depending upon maternal sociodemographic and economic characteristics and maternal characteristics during pregnancy. Children of African American women had almost twice the risk of infant mortality when compared with children of White women (AOR 2.12; 95% CI, 1.98–2.27; p<0.001). Infants of women with Bachelor’s degrees or higher were 89% less likely to die (AOR 1.89; 95% CI, 1.76–2.04; p<0.001) when compared to infants of women with education less than high school. Infants of maternal smokers were 75% more likely to die (AOR 1.75; 95% CI, 1.58–1.93; p<0.001) than infants of nonsmokers. Infants of women who were overweight and obese during pregnancy accounted for 55% of IMR over all women in the study. More than half of the infant deaths were to children of women with lower socioeconomic status; infants of WIC participants were 59% more likely to die (AOR 1.59; 95% CI, 1.52–1.67; p<0.001) than infants of non-WIC participants. With respect to infant predictors, infants born with LBW or PTB were more than six times (AOR 6.29; 95% CI, 5.90–6.70; p<0.001) and almost four times (AOR 3.95; 95% CI, 3.73–4.19; p<0.001) more likely to die than infants who had normal births, respectively. SGA and LGA infants were more than two times (AOR 2.03; 95% CI, 1.92–2.15; p<0.001) and 41% (AOR 1.41; 95% CI, 1.32–1.52; p<0.001) more likely to die than AGA infants, respectively.

**Conclusions:**

While the overall IMR in California is declining, wide disparities in death rates persist in different groups, and these disparities are increasing. Our data indicate that maternal sociodemographic and economic factors, as well as maternal prepregnancy obesity and smoking during pregnancy, have a prominent effect on IMR though no causality can be inferred with the current data. These predictors are not typically addressed by direct medical care. Infant factors with a major effect on IMR are birthweight and gestational age—predictors that are addressed by active medical services. The highest value interventions to reduce IMR may be social and public health initiatives that mitigate disparities in sociodemographic, economic and behavioral risks for mothers.

## Introduction

The infant mortality rate (IMR) is a standardized measurement of deaths in the first year of life per thousand live births. It is a well-recognized indicator of the general health of the population, and has been steadily declining in the US [1]. The IMR reflects broad socio-economic conditions and the educational status of the population, as well as quality and accessibility of medical services. IMR remains relatively high in the US when compared with other developed countries [2].

Even though significant improvements have been made in the quality and access to neonatal and infant care during the past decade, large educational, socioeconomic, racial, ethnic, geographic and behavioral disparities persist, and appear to be responsible for significant disparities in IMR among different subgroups. Certain maternal and infant characteristics have important associations with IMR, and this study attempted to quantify major maternal and infant predictors and trace associated mortality trends during the study period.

The neonatal mortality rate (NMR) is a standardized measurement of deaths in the first 27 days of life per thousand live births and has different significance. NMR reflects prevalence of antenatal abnormalities such as birth defects and intrauterine infections, as well as quality of neonatal care. This rate is less sensitive to educational and socioeconomic differences in the population.

Major infant predictors associated with NMR are birthweight and gestational age. Birthweight is a principal factor: both restriction in fetal growth and increased fetal growth are important factors associated with infant mortality [3]. Restrictions in fetal growth, also known as intrauterine growth restriction (IUGR) have been associated with many common complications of pregnancy [4]. IUGR may be identified during pregnancy by ultrasound, either with a lack of appropriate growth on serial measurements, or a measurement below a specific percentile, such as being below the 10th percentile for gestational age and gender [3, 5, 6]. When the sonographic assessment of intrauterine growth is not readily accessible, small-for-gestational-age (SGA) at birth may be used to reflect the degree of fetal growth restriction (FGR) [3, 5].

The SGA infant weighs below the 10th percentile for gestational age and gender within the reference population [9, 13, 14]. The appropriate-for-gestational-age (AGA) infant weighs between the 10^th^ and 90^th^ percentile and large-for-gestational age weighs above 90^th^ percentile for gestational age and gender within the reference population [8, 14]. SGA and large-for-gestational-age (LGA) infants are at increased risk of neonatal and infant mortality [7] with sequelae, extending beyond the perinatal period [7].

Preterm birth (PTB), defined as delivery at less than 37 weeks of gestation, occurs in 5 to 18% of pregnancies [8]. It is a leading cause of infant mortality and the second most common cause of mortality in infants and children under 5 years of age [9].

This retrospective study aimed to analyze birth cohort data from the linked infant birth-death files compiled by the California Department of Public Health for 2007–2015 to better clarify these maternal and infant predictors of infant deaths. The data collection and analysis give particular attention to the relative roles of maternal educational and socioeconomic factors, perinatal smoking and obesity, and to infant SGA and LGA in predicting infant mortality. Our hypothesis was that predictors of infant mortality are trending unevenly in the US, with definite improvements in categories strongly influenced by medical and scientific advances, but with minimal improvement in reduction of non-medical sociodemographic, economic and maternal behavioral disparities associated with negative infant outcomes.

## Materials and methods

### Data source

This retrospective study analyzed data from the linked birth and infant death files (birth cohorts) and live births from the Birth Statistical Master Files (BSMF) in California compiled by the California Department of Public Health (CDPH) for 2007–2015.

In the birth cohort files, information from death certificates for each cohort less than 365 days old in a given year is linked to the birth certificate [1]. Therefore, linked files include maternal indicators and birth outcomes from the birth certificate. Links to the death certificate adds age at death and underlying and multiple cause(s) of death. Almost 99% of infant deaths were linked to corresponding birth certificates [1]. Chances are extremely low for duplicate records for infant deaths.

The study was approved by the California Committee for the Protection of Human Subjects and by the CDPH Vital Statistics Advisory Committee. This study involved a retrospective analysis of anonymized clinical data and patient consent was waived by the Human Subjects Committee.

### Cohort selection

**Fig 1:**
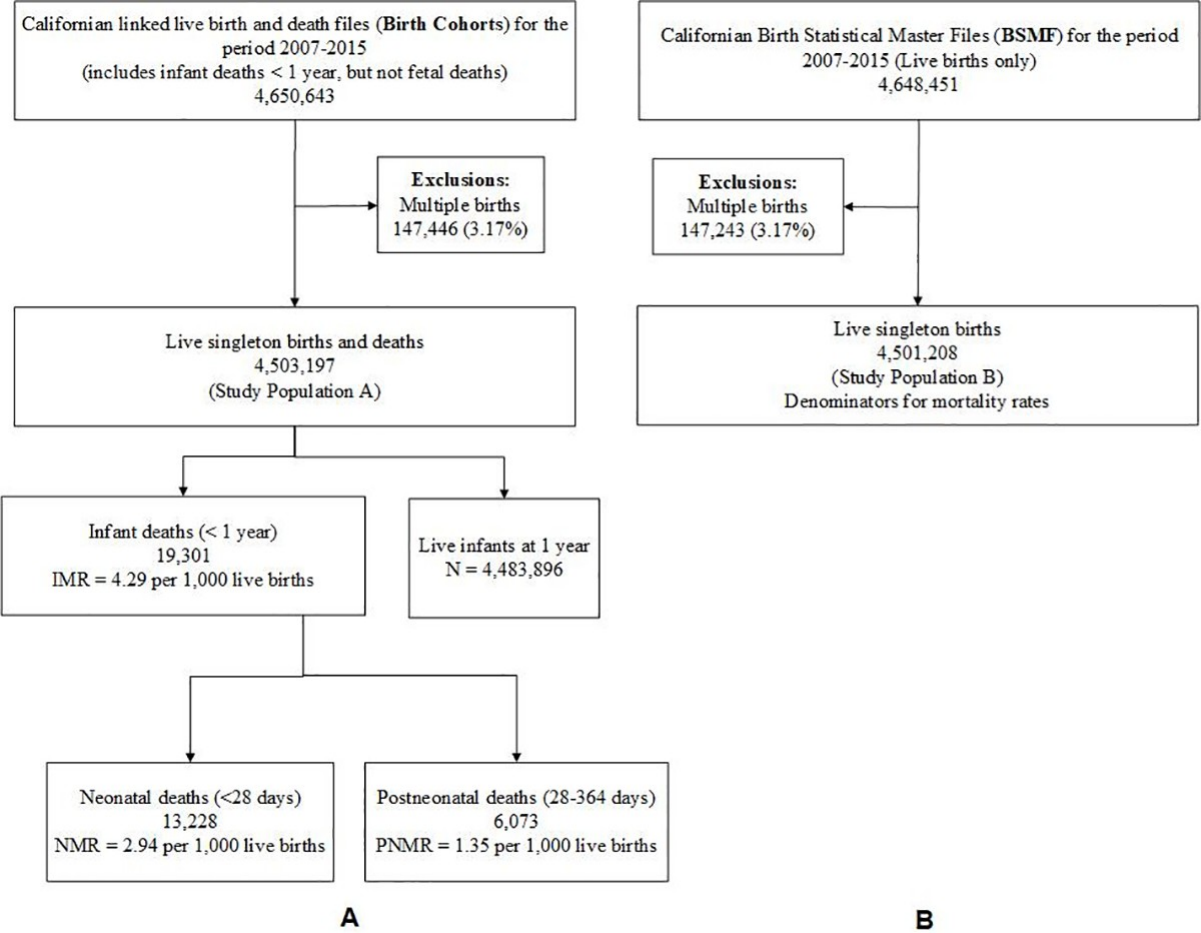
Screening criteria to identify study populations (A) Californian linked birth and infant death files (Birth Cohorts), 2007–2015, and (B) Californian live births from the Birth Statistical Master files (BSBF), 2007–2015, to study infant mortality rate (IMR), neonatal mortality rate (NMR), and postneonatal mortality rate (PNMR) per 1,000 live births for singleton births only in California for the period 2007–2015.

From the initial birth cohort of 4,650,643 infants, 147,446 (3.17%) multiple births were excluded. This left 4,503,197 singletons with 19,301 infant deaths as the primary study population during the nine-year study period 2007–2015, as shown in “Study Population A” in Fig 1.

**Fig 2:**
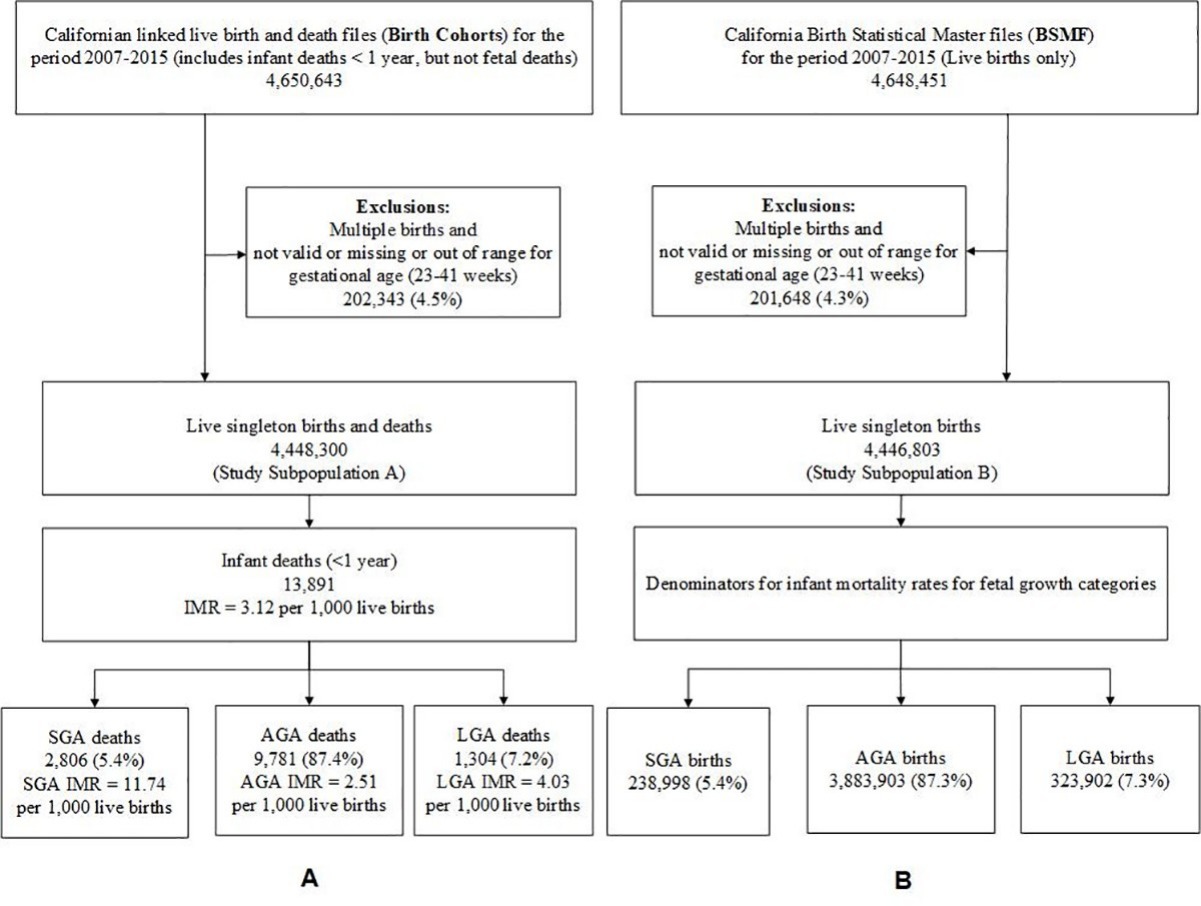
Screening criteria to identify study subpopulations (A) Californian linked birth and infant death files (Birth Cohorts), 2007–2015, and (B) Californian live births from the Birth Statistical Master files (BSBF), 2007–2015, to study infant mortality rate (IMR) by fetal growth (SGA (small-for-gestational-age), AGA (appropriate-for-gestational-age, and LGA (large-for-gestational-age) for singleton births only in California for the period 2007–2015.

A subpopulation to evaluate IMR in relation to fetal growth included singletons at 23–41 weeks of gestation [10]. This study subpopulation consisted of 4,448,300 births with 13,891 infant deaths during the study period (Study Subpopulation A in Fig 2), after excluding 54,897 records (1.22%).

The corresponding study population and study subpopulation with live births from the BSMF for the denominator were selected in the same manner. The denominator file consists of all live births in a given year and included 4,501,208 resident singleton live births, excluding 147,243 (3.17%) multiple births (Study Population B in Fig 1).

A subpopulation to study mortality rates for fetal growth included 4,446,803 singleton live births at 23–41 weeks of gestation (Study Subpopulation B in Fig 2).

Gestational age was derived from the obstetric estimate from ultrasound measurements, which is considered superior to data based on the date of the last menstrual period (LMP) [11]. Birth and infant death records in the birth cohort and live births from the BSMF were de-identified with respect to the individual women who gave birth and therefore, data included all individual pregnancies.

### Exposure variables

The revised gender-specific intrauterine growth curves developed for the determination of gestational age, based on the obstetric estimate (OE) and US population data [10], were used to identify SGA, appropriate-for-gestational-age (AGA), and LGA births in this study. A previously reported methodology was used to identify low birth weight (LBW), preterm birth (PTB), SGA, LGA, and cesarean delivery (CD) from the dataset. These factors were coded as dichotomous variables and indicated whether the infant was LBW, PTB, SGA, AGA, LGA, or CD [12, 13].

### Infant mortality

Three mortality rates, neonatal mortality, postneonatal mortality and infant mortality were assessed in this study using standard definitions (deaths between 0–27 days, 28 to 364 days and 0 to 364 days respectively per 1,000 live births.

### Covariates

Potential covariates analyzed in the multivariate model included the birth year, maternal sociodemographic characteristics, indicators of poverty, maternal prepregnancy obesity, and smoking during pregnancy.

The indicators of maternal sociodemographic status included educational level, maternal age, race, ethnicity, nativity and geographical region. Several factors were used to characterize maternal socioeconomic status: receipt of benefits from the federal Supplemental Nutrition Program for Women, Infants, and Children (WIC) and receipt of Medi-Cal. Use of Medi-Cal versus private insurance is crudely predictive of low and high patient incomes, respectively.

Data were analyzed on parity and whether women had prenatal care during the first trimester of pregnancy, which are recognized factors associated with health outcomes for the mother and infant [12, 13].

Maternal prepregnancy obesity and smoking during both first and second trimesters of pregnancy that affected health outcomes were described before [14, 15]. The maternal pre-pregnancy body mass index (BMI) (weight in kg/height in m^2^) was categorized using World Health Organization (WHO) criteria as: BMI <18.5 kg/m^2^, underweight; BMI 18.5–24.9 kg/m^2^, normal weight; BMI 25.0–29.9 kg/m^2^, overweight; BMI 30.0–34.9 kg/m^2^, class I obesity; BMI 35.0–39.9 kg/m^2^, class II obesity; and BMI > 40 kg.m^2^, class III obesity.

### Descriptive analysis

First, a descriptive analysis was undertaken of maternal sociodemographic characteristics, economic status, prepregnancy obesity, and maternal smoking during pregnancy. The descriptive analysis was based on the Study Population A of 4,503,197 births and deaths which included 19,301 infant deaths and Study Population B of 4,501,208 singleton live births (Fig 1). A simple linear regression analysis was applied to access the annual trend for infant mortality rates according to the study variables. Mortality rate was considered the dependent variable in the linear regression models. Cochran-Armitage trend testing was also used to assess for linear trend in neonatal, postneonatal, and infant mortality rates. We also examined those trends employing logistic regression models adjusting for maternal demographic characteristics, prepregnancy obesity, smoking during pregnancy, delivery mode, birth weight (grams) and gestational age (weeks).

Second, the primary infant factors, birthweight and gestational age, were assessed in relation to IM. For the main study variables of SGA and LGA, the descriptive analysis was based on the birth cohort Subpopulation A of 4,448,300 which included 13,891 infant deaths and Subpopulation B of 4,446,803 live births (Fig 2) during the study period of 2007–2015. The analysis was extended to study the IMRs based on all cohorts according to two different interactions, between maternal age and maternal race and ethnicity, and between maternal level of education and maternal race and ethnicity.

Third, caesarean deliveries were evaluated to assess association with IM.

### Statistical analysis

Data were analyzed using SAS version 9.3 software (SAS Institute Inc., Cary, NC, USA). Simple linear regression was used to examine yearly trends. Two-way analysis of variance (ANOVA) was performed to determine the significance of the mean infant mortality rate for different ethnic and educational groups. Tukey's honestly significant difference (HSD) test was used for multiple comparisons to determine significant differences between the means. Analysis using logistic regression modeling of the study cohorts was performed to identify associations between predictors of infant mortality, SGA, and LGA. Potential confounding variables were controlled using multivariate analysis. Firth’s penalized likelihood approach was used to address issues of small sample sizes, and to estimate bias, and conventional maximum likelihood and the Firth penalized-likelihood estimates were compared [16, 17]. The unconditional estimates and the Firth penalized-likelihood estimates should be similar when the sample size is adequate. Cases with missing data for the required variables were excluded from the analysis. The calculated adjusted odds ratios (AORs) with a 95% confidence interval (CI) and the p-values were presented. A p-value <0.05 was considered to be statistically significant.

## Results

### Infant mortality rates in California from 2007 to 2015

**Fig 3:**
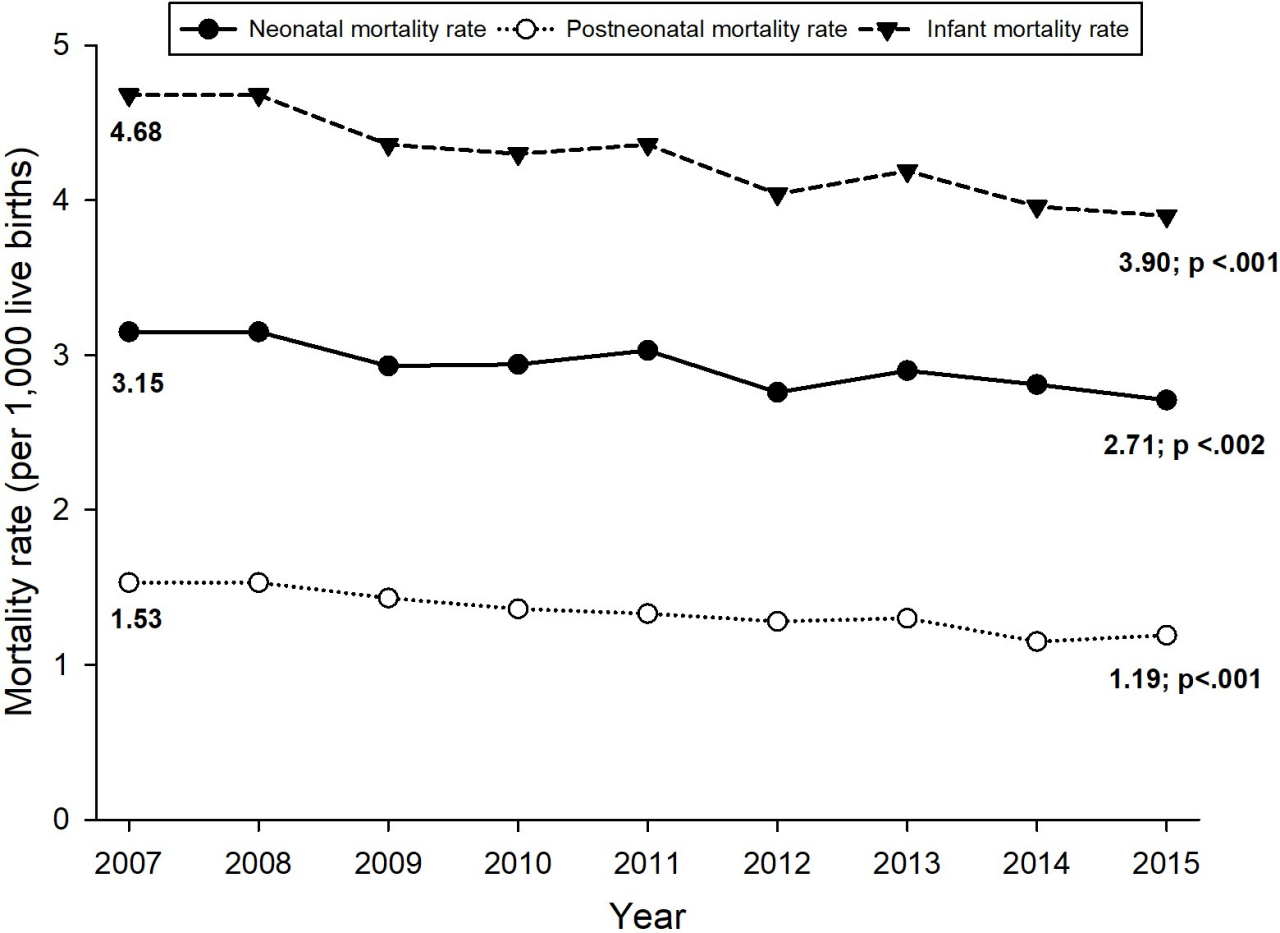
Infant, neonatal, and postneonatal mortalities (per 1,000 live births) for singleton births only in California for 2007 to 2015. p values indicate the significance of a linear regression. (Cochran-Armitage trend test also showed the declining trends in neonatal (p <0.001), postneonatal (p<0.001), and infant mortality rates (p<0.001) for singleton births from 2007 to 2015).

**Fig 4:**
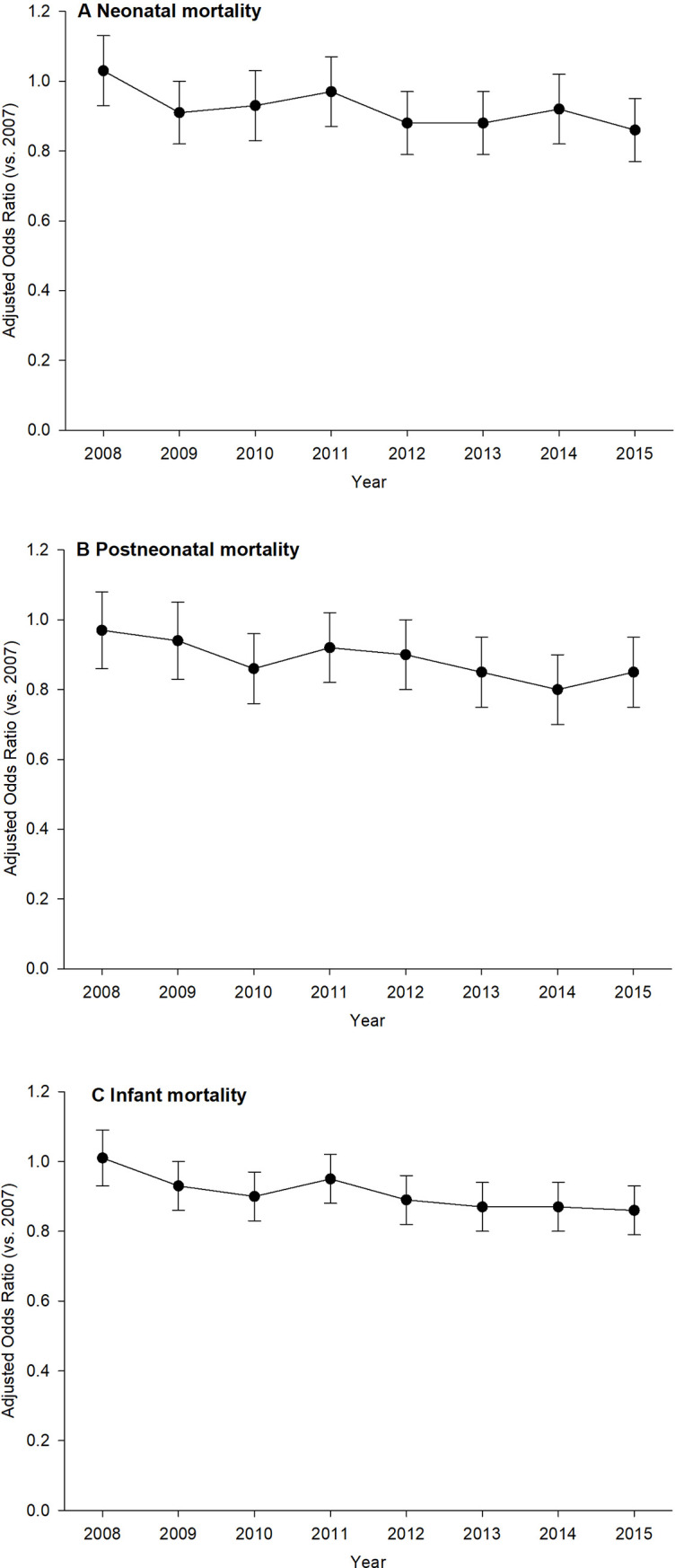
Adjusted odds ratio (with 95% confidence interval in error bars) for A) neonatal mortality (p = 0.014), B) postneonatal mortality (p = 0.008), and C) infant mortality (p <0.001), for singleton births in California for the period 2007–2015. Models were adjusted for maternal demographic characteristics, prepregnancy obesity, smoking during pregnancy, delivery mode, birth weight (grams) and gestational age (weeks). Year 2007 is the reference group. The selected model for the neonatal, postneonatal, and infant mortality showed a concordance index of, c = 0.886, 0.757, and c = 0.826, respectively.

The neonatal (3.15 in 2007 to 2.71 in 2015), postneonatal (1.53 in 2007 to 1.19 in 2015), and infant mortality (4.68 in 2007 to 3.90 in 2015) rates for singleton births decreased linearly from 2007 to 2015, with the significance of these regressions being p = 0.002, p <0.001, and p < .001, respectively (Fig 3). Cochran-Armitage trend test also showed the declining trends in neonatal (p <0.001), postneonatal (p<0.001), and infant mortality rates (p<0.001) for singleton births from 2007 to 2015. Adjusted logistic regression models also showed the declining trends in neonatal (p = 0.014), postneonatal (p = 0.008), and infant mortality rates (p <0.001) for singleton births from 2007 to 2015 (Fig 4). The performance characteristic measure for models neonatal, postneonatal, and infant mortality was indicated in foot notes of the Fig 4.

The mean maternal age in the selected population linearly increased significantly from 28.0 years in 2007 to 29.4 years in 2015 (p < .001).

The mean birth weight (grams) linearly decreased significantly from 3,333 grams in 2007 to 3,321 grams in 2015 by 11.9 grams (p = 0.013) while gestational age (in weeks) linearly increased significantly from 37.94 weeks in 2007 to 38.68 weeks in 2015 by 0.74 weeks (p < 0.012) during the study period from 2007 to 2015 (S1 Table).

### Maternal sociodemographic characteristics and economic status and IMR

#### Age

The total number of births and infant deaths with their percentages are shown in Table 1. Younger and older maternal ages were associated with infant mortality. Women <20 years of age gave birth to 7.8% of the infants and represented 10.9% of the total cases of infant mortality while women aged 40–54 years of age had 3.8% of births but represented 5.6% of the total cases of infant deaths (Table 1). Births to women <20 years of age and to those 40–54 years of age experienced 28% (AOR 1.28; 95% CI, 1.19–1.39; p<0.001) and 69% more infant deaths (AOR 1.69; 95% CI, 1.56–1.84; p<0.001), respectively, when compared with women of 30–34 years of age (Table 2). No significant change in the infant mortality rate occurred during the study period of 2007–2015 for younger women, <30 years of age (Table 1).

**Table 1:** Partition of live singleton births from the BSMF, infant deaths from the birth cohorts, and infant mortality rate by birth year (deaths from the birth cohorts and live births from the BSMF) for maternal and infant characteristics in California for the period 2007–2015.

Characteristic	Total live singleton births from BSMF ^a^	Infant deaths among singletons from birth cohorts ^a^	Infant mortality rate (per 1,000 live singleton births) ^b^									p value ^c^
			2007	2008	2009	2010	2011	2012	2013	2014	2015	
Overall			4.68	4.68	4.36	4.3	4.36	4.04	4.19	3.97	3.9	< .001
***Maternal age (years)***												
<20	349,801 (7.8)	2,078 (10.9)	6.4	6.22	5.89	5.56	5.89	6.2	6.33	5.37	4.95	0.064
20–24	936,464 (20.8)	4,494 (23.5)	4.85	5.13	4.7	4.69	5.1	4.56	4.78	4.73	4.55	0.158
25–29	1,203,344 (26.7)	4,733 (24.7)	4.3	4.11	3.79	4.08	3.6	3.79	4.01	3.71	3.94	0.158
30–34	1,181,474 (26.3)	4,146 (21.7)	3.85	3.74	3.57	3.79	3.83	3.27	3.31	3.21	3.11	0.003
35–39	658,610 (14.6)	2,612 (13.7)	4.54	4.72	4.22	3.66	3.85	3.8	3.75	3.71	3.46	0.002
40–54	170,738 (3.8)	1,066 (5.6)	6.61	6.35	7.23	5.66	7.19	5.02	6.1	6.15	5.97	0.003
***Maternal race and ethnicity***												
Hispanic	2,262,159 (51.2)	9,760 (52.3)	4.59	4.76	4.26	4.39	4.31	4.18	4.36	3.81	4.01	0.006
White	1,212,987 (27.5)	4,173 (22.4)	4.11	3.73	3.41	3.49	3.41	3.07	3.34	3.26	3.05	0.003
Asian	579,629 (13.1)	1,842 (9.9)	4.1	3.85	3.49	2.79	3.47	2.91	2.69	2.71	2.71	0.002
Pacific Islander	19,350 (0.4)	131 (0.7)	9.16	4.58	8.57	5.5	10.98	4.01	4.65	7.66	5.17	0.437
African American	233,560 (5.3)	2,163 (11.6)	10.39	11.14	9.2	8.88	8.87	8.66	8.41	9.17	8.19	0.012
Multiple race	91,909 (2.1)	474 (2.5)	5.18	5.34	5.9	4.44	4.39	5.02	5.37	5.32	5.46	0.893
American Indian	16,234 (0.4)	109 (0.6)	6.65	8.13	4.66	9.63	9.63	4.05	5.78	5.95	5.59	0.401
***Maternal education***												
Less than high school diploma	962,439 (22.2)	4,901 (28.1)	5.25	5.15	4.92	5.17	5.16	4.92	5.15	4.85	5.18	0.416
High school diploma	1,139,705 (26.3)	5,379 (30.8)	5.23	5.04	4.61	4.65	5.02	4.36	4.72	4.44	4.25	0.011
Some college or associate degree	1,082,385 (25.0)	4,482 (25.7)	4.41	4.45	4.29	4.15	4.14	3.93	4.1	3.96	3.9	< .001
Bachelor's degree or higher	1,142,505 (26.4)	2,703 (15.5)	2.9	2.8	2.47	2.14	2.31	2.33	2.23	2.08	2.14	0.004
***Maternal nativity***												
Foreign-born	1,848,786 (41.1)	7,019 (36.4)	4.18	4.13	3.88	3.71	3.82	3.7	3.69	3.36	3.43	< .001
United States-born	2,650,862 (58.9)	12,277 (63.6)	5.11	5.13	4.72	4.72	4.72	4.26	4.5	4.34	4.18	< .001
***Maternal demographic region***												
Central Coast	265,224 (5.9)	1,077 (5.6)	4.07	4.7	4.01	3.97	3.83	4.43	4.13	3.84	3.48	0.127
Greater Bay Area	780,152 (17.3)	2,636 (13.7)	3.94	3.37	3.61	3.54	3.35	3.17	3.2	3.17	2.99	0.002
Inland Empire	550,017 (12.2)	2,791 (14.5)	4.89	5.44	5.74	5.14	4.95	5.24	5.03	4.78	4.44	0.075
Los Angeles County	1,179,627 (26.2)	5,046 (26.1)	4.88	4.9	4.36	4.26	4.26	4.14	3.88	3.75	3.87	< .001
Northern and Sierra	139,234 (3.1)	709 (3.7)	4.91	5	4.68	5.28	5.65	4.84	4.96	5.62	4.92	0.483
Orange County	342,866 (7.6)	1,145 (5.9)	4.08	3.78	3.29	3.35	4.03	2.6	3.17	3.12	2.47	0.019
Sacramento area	245,037 (5.4)	1,070 (5.5)	4.74	4.59	4.33	4.12	4.1	4.21	4.09	4.37	4.68	0.56
San Diego area	418,154 (9.3)	1,590 (8.2)	4.57	4.39	3.94	3.88	3.9	3.14	3.73	3.47	3.13	0.001
San Joaquin Valley	580,897 (12.9)	3,237 (16.8)	5.73	5.99	5.11	5.44	5.89	4.95	6.2	5.11	5.72	0.795
***Source of prenatal care payment***												
Private	2,082,931 (50.2)	6,663 (39.6)	3.69	3.53	3.32	3.14	3.31	2.9	2.94	2.97	2.9	< .001
Medi-Cal	2,069,734 (49.8)	10,148 (60.4)	5.04	5.1	4.79	4.89	4.94	4.82	4.98	4.71	4.8	0.063
***WIC Participation***												
No	2,151,875 (47.8)	9,413 (48.8)	5.09	4.97	4.59	4.33	4.38	4	4.09	3.9	3.85	< .001
Yes	2,349,333 (52.2)	9,888 (51.2)	4.28	4.41	4.16	4.28	4.34	4.08	4.28	4.04	3.95	0.033
***First trimester prenatal care***												
No	747,742 (16.9)	3,722 (20.8)	5.44	5.17	5.04	4.64	5.36	5.18	5.05	4.66	4.16	< .001
Yes	3,666,738 (83.1)	14,152 (79.2)	4.14	4.18	3.88	3.96	3.85	3.64	3.77	3.77	3.64	0.043
***Parity***												
Primiparous	1,775,913 (39.5)	7,579 (40.1)	4.74	4.56	4.42	4.08	4.39	4.13	4.23	3.89	3.87	0.001
Multiparous (2–5)	2,629,967 (58.5)	10,611 (56.1)	4.37	4.33	4.04	4.23	4.14	3.79	3.88	3.72	3.74	< .001
Multiparous (6–12)	88,090 (2.0)	718 (3.8)	9.01	8.77	8.51	7.85	7.34	7.32	9.26	9.54	5.58	0.311
***Maternal smoking during both first and second trimesters***												
No	4,349,579 (98.4)	17,434 (96.2)	4.27	4.34	4.08	4.08	4.09	3.81	3.91	3.74	3.69	< .001
Yes	70,240 (1.6)	694 (3.8)	10.96	9.42	8.56	7.9	9.92	10.18	11.47	10.91	10.02	0.36
***Prepregnancy BMI***												
Underweight (≤18.5)	169,353 (4.0)	624 (3.8)	4.31	4.26	3.49	3.06	4.2	4.11	3.02	3.64	2.91	0.096
Normal (18.5–24.9)	2,058,902 (49.1)	6,807 (41.2)	3.52	3.58	3.33	3.21	3.34	3.3	3.3	3.22	2.89	0.007
Overweight (25.0–29.9)	1,083,749 (25.9)	4,394 (26.6)	4.47	4.24	4.13	4.28	4.17	3.86	3.99	3.7	3.65	< .001
Obese I (30.0–34.9)	532,167 (12.7)	2,606 (15.8)	5.17	5.63	5.21	4.81	4.72	4.51	4.89	4.63	4.55	0.011
Obese II (35.0–39.9)	221,449 (5.3)	1,262 (7.6)	5.88	5.99	5.82	6.12	5.94	5.12	5.47	5.4	5.63	0.07
Obese III (≥ 40)	125,693 (3.0)	820 (5.0)	7.06	6.54	5.79	7.98	6.4	5.65	7	5.9	6.53	0.527
***Child sex***												
Male	2,306,969 (51.3)	10,682 (55.5)	5.09	5.03	4.84	4.65	4.66	4.35	4.56	4.31	4.07	< .001
Female	2,194,186 (48.8)	8,581 (44.6)	4.23	4.31	3.85	3.91	4.03	3.69	3.79	3.59	3.7	0.004
***Delivery method***												
Vaginal delivery	3,094,184 (68.7)	11,373 (60.1)	3.99	3.97	3.9	3.72	3.68	3.52	3.65	3.39	3.18	< .001
Cesarean delivery	1,407,023 (31.3)	7,542 (39.9)	5.97	5.75	5.13	5.44	5.47	4.97	5.08	5.09	5.25	0.025

^a^ Percentage of characteristic given in parentheses.

^b^ The numerator was from the linked birth and infant death files (Birth Cohorts) and the denominator was from the California Birth Statistical Master Files (BSMF) which consist of all live births in a given year.

^c^ p value for linear regression.

**Table 2:** Crude and adjusted odds ratios (with 95% confidence intervals in parentheses) of infant mortality for singleton births for maternal and infant characteristics in California for the period 2007–2015.

Characteristic	Crude odds ratio		Adjusted odds ratio	
	OR (95% CI)	p value^a^	OR (95% CI)	p value^a^
***Birth year***				
2008	1.00 (0.95–1.06)	0.988	1.03 (0.96–1.11)	0.376
2009	0.93 (0.88–0.99)	0.014	0.94 (0.88–1.01)	0.085
2010	0.92 (0.87–0.97)	0.004	0.93 (0.87–1.00)	0.059
2011	0.93 (0.88–0.99)	0.014	0.97 (0.90–1.04)	0.386
2012	0.86 (0.81–0.92)	< .001	0.92 (0.86–0.99)	0.028
2013	0.90 (0.84–0.95)	< .001	0.93 (0.87–1.00)	0.056
2014	0.85 (0.80–0.90)	< .001	0.91 (0.84–0.98)	0.009
2015	0.83 (0.78–0.88)	< .001	0.90 (0.84–0.97)	0.007
2007 (ref)	Ref^b^		Ref^b^	
***Maternal age*, *years***				
< 20	1.70 (1.61–1.79)	< .001	1.28 (1.19–1.39)	< .001
20–24	1.37 (1.31–1.43)	< .001	1.09 (1.03–1.15)	0.004
25–29	1.12 (1.08–1.17)	< .001	1.01 (0.96–1.06)	0.729
35–39	1.13 (1.08–1.19)	< .001	1.14 (1.07–1.21)	< .001
40–54	1.78 (1.67–1.91)	< .001	1.69 (1.56–1.84)	< .001
30–34 (ref)	Ref^b^		Ref^b^	
***Maternal race/ethnicity***				
African American	2.71 (2.57–2.85)	< .001	2.12 (1.98–2.27)	< .001
American Indian	1.95 (1.61–2.36)	< .001	1.36 (1.07–1.73)	0.013
Asian	0.92 (0.88–0.98)	0.005	1.37 (1.28–1.47)	< .001
Hispanic	1.26 (1.21–1.30)	< .001	1.13 (1.08–1.19)	< .001
Multiple Race	1.50 (1.37–1.65)	< .001	1.42 (1.27–1.59)	< .001
Pacific Islander	1.98 (1.66–2.35)	< .001	1.76 (1.42–2.18)	< .001
White (ref)	Ref^b^		Ref^b^	
***Maternal education***				
< High school	2.17 (2.07–2.28)	< .001	1.89 (1.76–2.04)	< .001
High school diploma	2.00 (1.91–2.09)	< .001	1.71 (1.61–1.83)	< .001
Some college/associate degree	1.75 (1.67–1.84)	< .001	1.53 (1.44–1.62)	< .001
Bachelor's degree or higher (ref)	Ref^b^		Ref^b^	
***Maternal nativity***				
United States–born	1.22 (1.19–1.26)	< .001	1.17 (1.12–1.22)	< .001
Foreign–born (ref)	Ref^b^		Ref^b^	
***Maternal demographic region***				
Central Coast	1.07 (0.99–1.16)	0.094	1.19 (1.08–1.31)	0.001
Greater Bay Area	0.89 (0.84–0.95)	< .001	1.01 (0.93–1.10)	0.771
Inland Empire	1.34 (1.26–1.42)	< .001	1.40 (1.29–1.52)	< .001
Los Angeles County	1.13 (1.06–1.19)	< .001	1.15 (1.07–1.25)	< .001
Northern and Sierra	1.34 (1.22–1.46)	< .001	1.31 (1.17–1.47)	< .001
Orange County	0.88 (0.81–0.95)	0.001	1.09 (0.99–1.20)	0.067
Sacramento Area	1.15 (1.06–1.24)	0.001	1.23 (1.12–1.36)	< .001
San Joaquin Valley	1.47 (1.38–1.56)	< .001	1.51 (1.40–1.64)	< .001
San Diego Area (ref)	Ref^b^		Ref^b^	
***Source of prenatal care payment***				
Medi–Cal (Public)	1.54 (1.49–1.58)	< .001	1.44 (1.37–1.52)	< .001
Private insurance (ref)	Ref^b^		Ref^b^	
***WIC food recipients***				
No	1.04 (1.01–1.07)	0.008	1.59 (1.52–1.67)	< .001
Yes (ref)	Ref^b^		Ref^b^	
***First-trimester prenatal care***				
No	1.29 (1.25–1.34)	< .001	0.99 (0.94–1.03)	0.617
Yes (ref)	Ref^b^		Ref^b^	
***Parity***				
Primiparous	1.06 (1.03–1.09)	< .001	1.10 (1.05–1.14)	< .001
Multiparous 6–12	2.03 (1.88–2.19)	< .001	1.47 (1.33–1.62)	< .001
Multiparous 2–5 (ref)	Ref^b^		Ref^b^	
***Maternal smoking during both first and second trimesters***				
Yes	2.48 (2.30–2.67)	< .001	1.75 (1.58–1.93)	< .001
No (ref)	Ref^b^		Ref^b^	
***Maternal prepregnancy BMI***				
Underweight, <18.5	1.12 (1.03–1.21)	0.010	1.17 (1.07–1.28)	0.001
Overweight, 25.0–29.9	1.23 (1.18–1.28)	< .001	1.14 (1.10–1.20)	< .001
Obese I–30.0–34.9	1.48 (1.42–1.55)	< .001	1.28 (1.21–1.34)	< .001
Obese II, 35.0–39.9	1.73 (1.63–1.84)	< .001	1.50 (1.40–1.61)	< .001
Obese III, ≥ 40	1.98 (1.84–2.13)	< .001	1.62 (1.50–1.76)	< .001
Normal, 18.5–24.9 (ref)	Ref^b^		Ref^b^	
***Child sex***				
Male	1.19 (1.15–1.22)	< .001	1.17 (1.13–1.21)	< .001
Female	Ref^b^		Ref^b^	
***Delivery method***				
Cesarean delivery	1.46 (1.42–1.50)	< .001	1.51 (1.46–1.57)	< .001
Vaginal delivery	Ref^b^		Ref^b^	

Abbreviations: OR: odds ratio; AOR: adjusted odds ratio; CI: confidence interval; BMI: body mass index.

^a^ p value for χ^2^ test.

^b^ Ref = Reference group.

Infant mortality and live singleton births defined in Fig 1.

#### Race and ethnicity

Hispanic women in California gave birth to 51% of the infants and represented 52.3% of the total cases of infant mortality. African American women in California gave birth to 5.3% of the infants but represented 11.6% of the total cases of infant mortality. However, Asian women gave birth to 13.1% of the infants but represented 9.9% of infant mortality. The infant mortality rate of births to Asian women significantly declined during the study period (p <0.001) and showed the lowest infant mortality rate of 2.71% in 2015 (Table 1). African American women had a significantly increased risk of infant mortality when compared with White women, with almost twice the risk (AOR 2.12; 95% CI, 1.98–2.27; p<0.001) (Table 2).

#### Education

Infants of mothers who had less than a high school education and who had 22.1% of the total births represented 28.1% of the total cases of infant mortality. However, 26.4% of the births to women with at least a bachelor’s degree represented 15.5% of the total cases of infant mortality. Women who had less than a high school education were 89% more likely to experience infant deaths (AOR 1.89; 95% CI, 1.76–2.04; p<0.001) when compared with women who had a bachelor’s degree or higher (Table 2).

#### Maternal age, race and ethnicity and infant mortality

**Fig 5:**
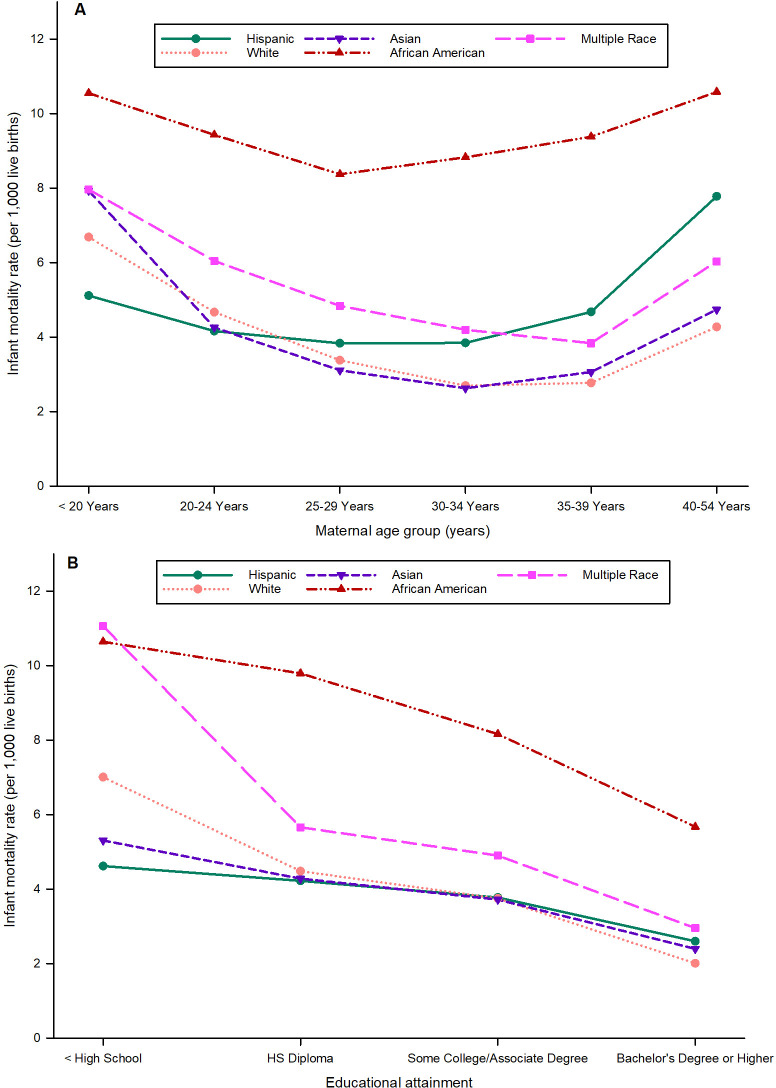
Unadjusted infant mortality rates (IMRs) in California for singleton births only for the period 2007–2015 for (A) maternal age and maternal race and ethnicity, and (B) maternal education and maternal race and ethnicity.

The association between maternal age and the race and ethnic groups by unadjusted IMR is displayed in Fig 5A. Women at the extremes of age, in the younger and older age groups, had an increased risk of infant mortality. African American women had higher IMRs compared with other races and ethnicities in each maternal age group studied (Fig 5A).

#### Maternal education level, race and ethnicity and infant mortality

The unadjusted IMRs were increased, and racial and ethnic disparities were greater when women had poor educational attainment in all races and ethnic groups but were dramatically higher for births to African American and mixed-race women (Fig 5B). IMRs were significantly lower (p <0.001), and disparities were significantly less (p <0.001) for women with higher education to the level of at least a bachelor’s degree (S2 Table). Even among educated mothers, African-American race was associated with higher IMR.

#### Maternal geographic region

**Fig 6:**
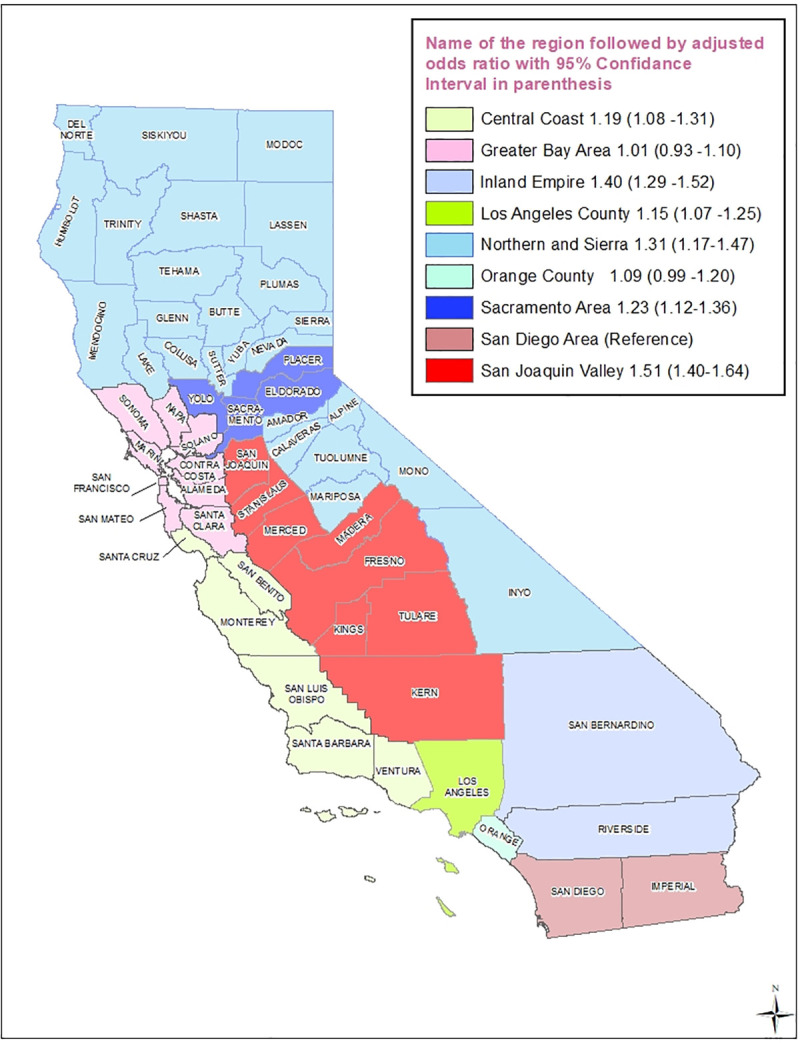
Adjusted odds ratio (with 95% confidence interval in parenthesis) for infant mortality for singleton births only for each demographic region identified on a map of California for the period 2007–2015. San Diego area is the reference group.

There was wide geographic disparity for infant mortality in California. Almost 13% of births occurred in San Joaquin Valley, a geographical area that had 16.8% of the total infant deaths in California (Table 1). In the rural San Joaquin Valley region, women were 51% (AOR 1.51; 95% CI, 1.40–1.64; p<0.001) more likely to experience infant deaths when compared to women in the San Diego area, which was an urban county (Table 2 and Fig 6).

### Maternal smoking and infant mortality

Maternal smokers were 75% more likely to have an infant death when compared with births to nonsmokers during the study period of 2007–2015 (AOR 1.75; 95% CI, 1.58–1.93; p<0.001).

### Maternal prepregnancy obesity

Using births to women of normal weight as the reference where the risk of infant mortality was lowest, infant mortality increased with increasing maternal body mass index (BMI). Infant mortality increased by 14% in overweight women (AOR 1.14; 95% CI, 1.10–1.20; p<0.001), by 28% in women (AOR 1.28; 95% CI, 1.21–1.34; p<0.001) with class I obesity, by 50% in women with class II obesity (AOR 1.50; 95% CI, 1.40–1.61; p<0.001), and by 62% in women with class III obesity (AOR 1.62; 95% CI, 1.50–1.76; p<0.001) (Table 2). Births to women with underweight were also vulnerable to infant deaths by 17% (AOR 1.17; 95% CI, 1.07–1.28; p<0.001).

### Infant factors

The rationale for adjusting for maternal and infant characteristics was that the results shown in Table 2 demonstrated that those characteristics were significantly associated with infant mortality. Moreover, LBW, PTB, SGA, and LGA were well known predictors for infant mortality.

The final model for the infant mortality was selected employing the performance characteristic measure concordance index (or area under the curve). Concordance index of selected models for infant, neonatal, and postneonatal mortality were c = 0.815, c = 0.873, and c = 0.758, respectively.

#### LBW and infant mortality

**Fig 7:**
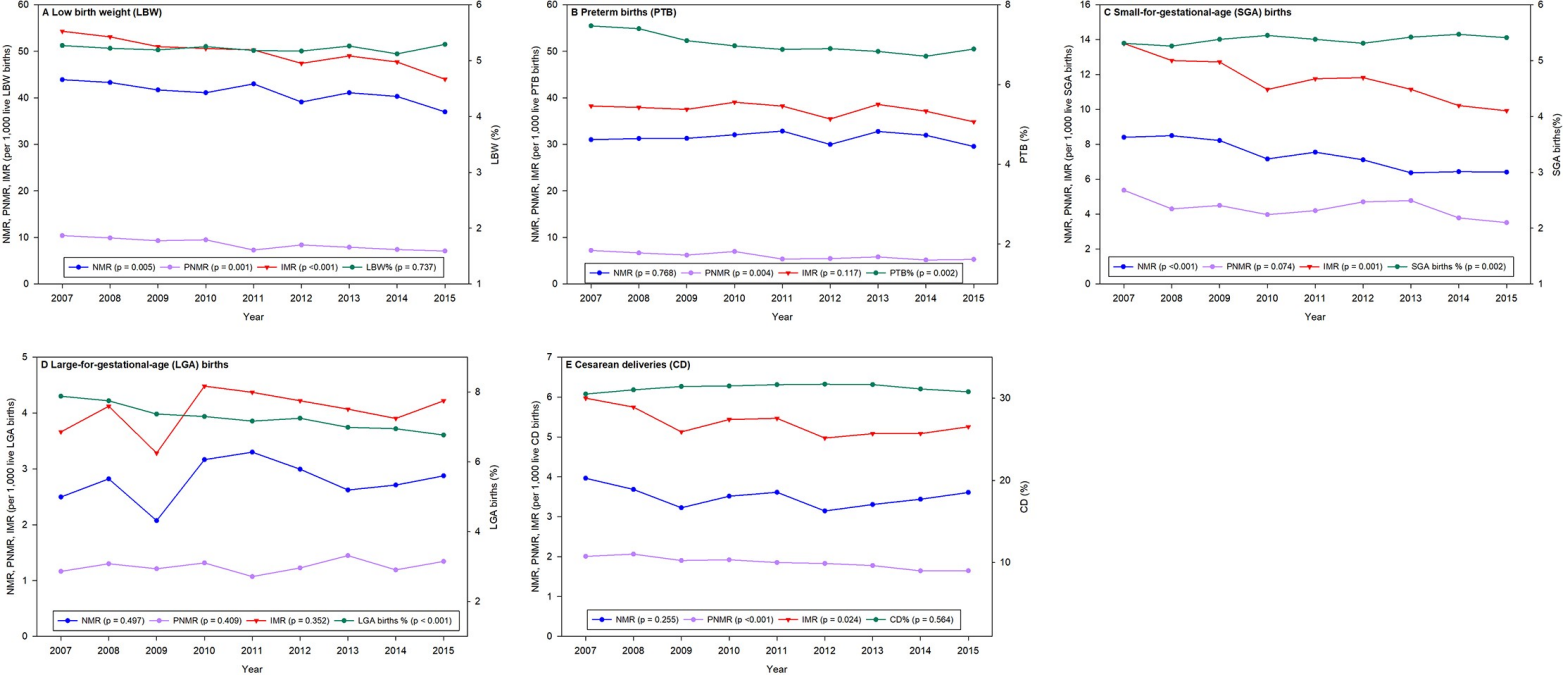
Neonatal mortality rate (NMR), postneonatal mortality rate (PNMR), and infant mortality rate (IMR) for singleton births only in California for 2007 to 2015 for A) LBW, B) PTB, C) SGA births, and D) LGA births and E) CD births. p values indicate the significance of a linear regression. Sub heddings: A. Low birth weight (LBW); B. Preterm births (PTB); C. Small-for-gestational-age (SGA) births; D. Large-for-gestational-age (LGA) births; E. Cesarean deliveries (CD).

LBW births were more than six times (AOR 6.29; 95% CI, 5.90–6.70; p<0.001) more likely to result in infant deaths compared with those infants who had normal birth weights (Table 4). While the rate of LBW was stable, the neonatal, postneonatal, and infant mortality associated with LBW linearly decreased significantly during the study period (Fig 7A).

#### PTB and infant mortality

PTB births were almost four times (AOR 3.95; 95% CI, 3.73–4.19; p<0.001) more likely to result in infant deaths compared with those infants who had normal births for PTB (Table 4). In contrast, the PTB rate linearly decreased significantly, but neonatal and infant mortality associated with PTB did not linearly decrease significantly during the study period (Fig 7B). However, postneonatal mortality linearly decreased significantly during the study period.

### Intrauterine growth and infant mortality

S1 Table shows the significance of linear trends and Table 3 shows the total number and prevalence in percentage of singleton births, infant deaths and mortality rates for the three main categories of intrauterine growth stages analyzed according to each variable studied in the subpopulation. The percentage of SGA, AGA, and LGA during the study period were 5.37% (n = 238,998), 87.34% (n = 3,883,903), and 7.28% (n = 323,902), respectively. The SGA birth did not change significantly from 5.31 in 2007 to 5.41 in 2015 (p = 0.069). However, AGA births increased from 86.81 in 2007 to 87.82 in 2015 (p<0.001), while LGA births declined 7.88 in 2007 to 6.77 in 2015 (p<0.001).

**Table 3:** Number of live singleton births and infant deaths and infant mortality rate by SGA, AGA, and LGA overall and for maternal and infant characteristics of study subpopulations in California for the period 2007–2015^a^.

Characteristic	Live singleton births ^b^				Infant deaths ^b^				Infant mortality rate (per 1,000 live singleton births)		
	Total	SGA	AGA	LGA	Total	SGA	AGA	LGA	SGA	AGA	LGA
**Overall**	4,446,803	238,998 (5.38)	3,883,903 (87.34)	323,902 (7.28)	13,891	2,806 (20.20)	9,781 (70.41)	1,304 9.39)	11.74	2.52	4.03
***Maternal age (years)***											
<20	344,539 (7.8)	26,433 (11.06)	303,930 (7.83)	14,176 (4.38)	1,569 (11.3)	306 (10.91)	1,151 (11.77)	112 (8.60)	11.58	3.79	7.90
20–24	923,629 (20.8)	55,939 (23.41)	813,689 (20.95)	54,001 (16.68)	3,327 (24.0)	620 (22.10)	2,437 (24.92)	270 (20.72)	11.08	3.00	5.00
25–29	1,189,348 (26.8)	59,991 (25.1)	1,041,322 (26.81)	88,035 (27.18)	3,437 (24.8)	650 (23.17)	2,453 (25.08)	334 (25.63)	10.83	2.36	3.79
30–34	1,168,779 (26.3)	56,032 (23.45)	1,018,336 (26.22)	94,411 (29.15)	2,897 (20.9)	567 (20.21)	2,025 (20.70)	305 (23.41)	10.12	1.99	3.23
35–39	651,472 (14.7)	31,523 (13.19)	562,196 (14.48)	57,753 (17.83)	1,847 (13.3)	411 (14.65)	1,232 (12.60)	204 (15.66)	13.04	2.19	3.53
40–54	168,726 (3.8)	9,059 (3.79)	144,175 (3.71)	15,492 (4.78)	812 (5.9)	251 (8.95)	483 (4.94)	78 (5.99)	27.71	3.35	5.03
***Maternal race and ethnicity***											
Hispanic	2,236,021 (51.2)	114,125 (48.69)	1,953,674 (51.25)	168,222 (52.95)	7,027 (52.1)	1,487 (54.29)	4,860 (51.21)	680 (54.23)	13.03	2.49	4.04
White	1,196,042 (27.4)	49,197 (21.01)	1,042,804 (27.37)	104,041 (32.76)	3,200 (23.7)	605 (22.09)	2,272 (23.94)	323 (25.76)	12.30	2.18	3.10
Asian	575,716 (13.2)	42,812 (18.26)	510,481 (13.39)	22,423 (7.06)	1,343 (10.0)	295 (10.77)	947 (9.98)	101 (8.05)	6.89	1.86	4.50
Pacific Islander	19,050 (0.4)	890 (0.38)	15,639 (0.41)	2,521 (0.79)	99 (0.7)	14 (0.51)	71 (0.75)	14 (1.12)	15.73	4.54	5.55
African American	229,958 (5.3)	21,358 (9.12)	196,596 (5.16)	12,004 (3.78)	1,359 (10.1)	253 (9.24)	1,018 (10.73)	88 (7.02)	11.85	5.18	7.33
Multiple race	90,762 (2.1)	5,150 (2.2)	78,817 (2.07)	6,795 (2.14)	366 (2.7)	72 (2.63)	256 (2.70)	38 (3.03)	13.98	3.25	5.59
American Indian	15,947 (0.4)	795 (0.34)	13,509 (0.36)	1,643 (0.52)	90 (0.7)	13 (0.47)	67 (0.71)	H10 (0.80)	16.35	4.96	6.09
***Maternal education level***											
< high school diploma	949,310 (22.2)	53,926 (23.4)	824,682 (21.96)	70,702 (22.59)	3,727 (28.9)	802 (30.60)	2,561 (28.13)	364 (30.69)	14.87	3.11	5.15
High school diploma	1,125,180 (26.3)	63,072 (27.58)	980,299 (26.28)	81,809 (26.29)	3,905 (30.3)	758 (28.92)	2,794 (30.69)	353 (29.76)	12.02	2.85	4.31
Some college or associate degree	1,070,955 (25.0)	55,302 (24.19)	933,343 (25.02)	82,310 (26.45)	3,239 (25.1)	648 (24.72)	2,308 (25.35)	283 (23.86)	11.72	2.47	3.44
Bachelor's degree or higher	1,131,307 (26.5)	56,822 (24.84)	997,707 (26.74)	76,778 (24.67)	2,040 (15.8)	413 (15.76)	1,441 (15.83)	186 (15.68)	7.27	1.44	2.42
***Maternal nativity***											
Foreign-born	1,828,355 (41.1)	101,617 (42.53)	1,601,769 (41.25)	124,969 (38.6)	4,999 (36.0)	1,157 (41.25)	3,336 (34.11)	506 (38.80)	11.39	2.08	4.05
United States-born	2,617,055 (58.9)	137,307 (57.47)	2,280,932 (58.75)	198,816 (61.4)	8,889 (64.0)	1,648 (58.75)	6,443 (65.89)	798 (61.20)	12.00	2.82	4.01
***Maternal demographic region***											
Central Coast	262,401 (5.9)	12,593 (5.27)	229,235 (5.9)	20,573 (6.35)	794 (5.7)	166 (5.92)	553 (5.65)	75 (5.75)	13.18	2.41	3.65
Greater Bay Area	770,874 (17.3)	42,207 (17.66)	672,803 (17.32)	55,864 (17.24)	1,960 (14.1)	351 (12.51)	1,408 (14.40)	201 (15.41)	8.32	2.09	3.60
Inland Empire	544,895 (12.3)	28,670 (12)	477,053 (12.28)	39,172 (12.1)	1,970 (14.2)	410 (14.61)	1,373 (14.04)	187 (14.34)	14.30	2.88	4.77
Los Angeles County	1,166,697 (26.2)	67,968 (28.44)	1,022,173 (26.32)	76,556 (23.64)	3,537 (25.5)	743 (26.48)	2,481 (25.37)	313 (24.00)	10.93	2.43	4.09
Northern and Sierra	136,229 (3.1)	6,942 (2.92)	117,741 (3.04)	11,546 (3.57)	560 (4.0)	101 (3.60)	412 (4.21)	47 (3.60)	14.55	3.50	4.07
Orange County	339,959 (7.7)	17,422 (7.29)	299,035 (7.7)	23,502 (7.25)	872 (6.3)	193 (6.88)	603 (6.17)	76 (5.83)	11.08	2.02	3.23
Sacramento area	241,770 (5.4)	12,146 (5.08)	208,932 (5.38)	20,692 (6.39)	762 (5.5)	141 (5.02)	553 (5.65)	68 (5.21)	11.61	2.65	3.29
San Diego area	414,141 (9.3)	20,159 (8.43)	362,615 (9.34)	31,367 (9.69)	1,082 (7.8)	221 (7.88)	751 (7.68)	110 (8.44)	10.96	2.07	3.51
San Joaquin Valley	569,837 (12.8)	30,891 (12.92)	494,316 (12.73)	44,630 (13.78)	2,354 (17.0)	480 (17.11)	1,647 (16.84)	227 (17.41)	15.54	3.33	5.09
***Source of prenatal care payment***											
Private	2,062,704 (50.2)	101,081 (45.93)	1,807,251 (50.37)	154,372 (51.58)	4,869 (38.7)	968 (37.77)	3,432 (38.73)	469 (39.95)	9.58	1.90	3.04
Medi-Cal	2,044,262 (49.8)	119,003 (54.07)	1,780,336 (49.63)	144,923 (48.42)	7,729 (61.4)	1,595 (62.23)	5,429 (61.27)	705 (60.05)	13.40	3.05	4.86
***WIC Participation***											
No	2,122,353 (47.7)	106,872 (44.72)	1,859,338 (47.88)	156,143 (48.22)	6,089 (43.83)	1,231 (43.87)	4,259 (43.54)	599 (45.94)	11.52	2.29	3.84
Yes	2,324,450 (52.3)	132,126 (55.28)	2,024,565 (52.12)	167,759 (51.78)	7,802 (56.17)	1,575 (56.13)	5,522 (56.46)	705 (54.06)	11.92	2.73	4.20
***First trimester prenatal care initiation***											
No	735,420 (16.8)	45,531 (19.47)	639,288 (16.76)	50,601 (15.91)	2,929 (22.00)	564 (20.92)	2,116 (22.53)	249 (19.97)	12.39	3.31	4.92
Yes	3,631,050 (83.2)	188,361 (80.53)	3,175,248 (83.24)	267,441 (84.09)	10,404 (78.00)	2,132 (79.08)	7,274 (77.47)	998 (80.03)	11.32	2.29	3.73
***Parity***											
Primiparous	1,750,945 (39.4)	127,648 (53.49)	1,534,236 (39.55)	89,061 (27.54)	5,215 (37.7)	1,043 (37.37)	3,752 (38.56)	420 (32.36)	8.17	2.45	4.72
Multiparous (2–5)	2,603,421 (58.6)	106,923 (44.81)	2,272,526 (58.59)	223,972 (69.26)	8,085 (58.5)	1,623 (58.15)	5,652 (58.09)	810 (62.40)	15.18	2.49	3.62
Multiparous (6–12)	86,482 (2.0)	4,045 (1.7)	72,086 (1.86)	10,351 (3.2)	519 (3.8)	125 (4.48)	326 (3.35)	68 (5.24)	30.90	4.52	6.57
***Maternal smoking during both first and second trimesters***											
No	4,302,407 (98.4)	227,492 (97.11)	3,760,695 (98.48)	314,220 (98.76)	12,870 (95.7)	2,619 (96.00)	9,020 (95.49)	1,231 (96.78)	11.51	2.40	3.92
Yes	68,795 (1.6)	6,752 (2.89)	58,090 (1.52)	3,953 (1.24)	576 (4.3)	109 (4.00)	426 (4.51)	41 (3.22)	16.14	7.33	10.37
***Prepregnancy body mass index (kg/m***^***2***^***)***											
Underweight (≤18.5)	167,857 (4.0)	16,079 (7.24)	147,498 (4.07)	4,280 (1.43)	512 (4.1)	123 (4.92)	348 (3.97)	41 (3.51)	7.65	2.36	9.58
Normal (18.5–24.9)	2,038,888 (49.1)	119,494 (53.85)	1,816,444 (50.07)	102,950 (34.29)	5,355 (43.1)	1,164 (46.56)	3,773 (43.08)	418 (35.82)	9.74	2.08	4.06
Overweight (25.0–29.9)	1,072,955 (25.9)	50,017 (22.53)	935,071 (25.77)	87,867 (29.26)	3,278 (26.4)	643 (25.72)	2,317 (26.45)	318 (27.25)	12.86	2.48	3.62
Obese I (30.0–34.9)	526,856 (12.7)	22,665 (10.21)	447,606 (12.34)	56,585 (18.84)	1,839 (14.8)	334 (13.36)	1,302 (14.86)	203 (17.40)	14.74	2.91	3.59
Obese II (35.0–39.9)	219,230 (5.3)	8,835 (3.98)	181,335 (5)	29,060 (9.68)	875 (7.0)	137 (5.48)	626 (7.15)	112 (9.60)	15.51	3.45	3.85
Obese III (≥ 40)	124,301 (3.0)	4,828 (2.18)	99,955 (2.76)	19,518 (6.5)	567 (4.6)	99 (3.96)	393 (4.49)	75 (6.43)	20.51	3.93	3.84
***Child sex***											
Male	2,279,051 (51.3)	127,148 (53.2)	1,987,535 (51.17)	164,368 (50.74)	7,765 (55.9)	1,524 (54.31)	5,522 (56.46)	719 (55.14)	11.99	2.78	4.37
Female	2,167,752 (48.8)	111,850 (46.8)	1,896,368 (48.83)	159,534 (49.26)	6,126 (44.1)	1,282 (45.69)	4,259 (43.54)	585 (44.86)	11.46	2.25	3.67
***Delivery method***											
Vaginal delivery	3,055,589 (68.71)	160,471 (67.15)	2,716,272 (69.94)	178,846 (55.22)	7,055 (50.8)	1,206 (42.98)	5,330 (54.49)	519 (39.80)	7.52	1.96	2.90
Cesarean delivery	1,391,213 (31.29)	78,527 (32.85)	1,167,630 (30.06)	145,056 (44.78)	6,836 (49.2)	1,600 (57.02)	4,451 (45.51)	785 (60.20)	20.38	3.81	5.41

Abbreviations: SGA, small-for-gestational-age; AGA, appropriate-for-gestational-age; LGA, large-for-gestational-age.

^a^ Screening criteria to identify Study Subpopulations defined in Fig 2.

^b^ Percentage of overall total or of characteristic given in brackets.

The associations for the unadjusted rates of SGA and LGA between maternal age groups and educational attainment by race and ethnic groups is displayed in S1 and S2 Figs, respectively.

S3 and S4 Tables illustrate crude and adjusted odds ratios of each variable studied for SGA and LGA births during the study period, respectively.

The infant mortality rates of SGA, AGA, and LGA were 11.74, 2.51, and 4.93 per 1,000 live births over the study period from 2007 to 2015, respectively.

SGA births had an increased risk of neonatal (AOR, 5.14; 95% CI, 4.82–5.48), postneonatal (AOR, 3.76; 95% CI, 3.48–4.07) and infant mortality (AOR, 4.53; 95% CI, 4.31–4.76), respectively, when compared with AGA births (Table 4).

**Table 4:** Adjusted odds ratio (AOR) for neonatal, post neonatal, and infant mortality by LBW, PTB, SGA and LGA from the multivariate logistic regression for women with live singleton births in the Study Subpopulation.

Variable	Neonatal mortality		Post neonatal mortality		Infant mortality	
	AOR (95% CI)	p value^a^	AOR (95% CI)	p value^a^	AOR (95% CI)	p value^a^
Low birth weight (LBW)^b^	9.51 (8.74–10.36)	< .001	3.60 (3.26–3.98)	< .001	6.29 (5.90–6.70)	< .001
Preterm birth (PTB)^c^	5.54 (5.11–6.00)	< .001	2.47 (2.25–2.71)	< .001	3.95 (3.73–4.19)	< .001
Small-for-gestational-age (SGA)^d^	2.02 (1.88–2.18)	< .001	2.13 (1.95–2.33)	< .001	2.03 (1.92–2.15)	< .001
Large-for-gestational-age (LGA)^e^	1.88 (1.72–2.06)	< .001	ns	< .001	1.41 (1.32–1.52)	< .001

Results are presented as AOR (95% CI) for neonatal, post neonatal, and infant mortalities by presence of LBW, PTB, SGA and LGA. Reference group consisted of absences of LBW, PTB, SGA and LGA, respectively.

^a^ p value for χ^2^ test; ns = not significant.

Multivariate logistic regression model adjusted for:

^b^ Maternal sociodemographic characteristics, prepregnancy BMI and smoking during pregnancy (birth year, maternal age, maternal race and ethnicity, maternal education, maternal nativity, maternal demographic region, source of prenatal care payment, WIC participation, first-trimester prenatal care initiation, parity, maternal smoking, and maternal prepregnancy body mass index), delivery mode, PTB, SGA, and LGA.

^c^ Maternal sociodemographic characteristics, prepregnancy BMI and smoking during pregnancy, delivery mode, LBW, SGA, and LGA.

^d^ Maternal sociodemographic characteristics, prepregnancy BMI and smoking during pregnancy, delivery mode, LBW, PTB, and LGA.

^e^ Maternal sociodemographic characteristics, prepregnancy BMI and smoking during pregnancy, delivery mode, LBW, PTB, and SGA.

Analyses were calculated based on Infant mortality and live singleton births defined in Fig 1.

LGA births had a 50%, 24%, and 24% greater risk for neonatal, postneonatal and infant mortality, respectively, when compared with AGA births (Table 4).

The rate of SGA was relatively stable; while the neonatal and infant mortality associated with SGA linearly decreased significantly during the study period (Fig 7C). However, postneonatal mortality did not linearly decrease significantly during the study period.

In contrast, LGA rate linearly decreased significantly, but neonatal, postneonatal, and infant mortality associated with LGA did not decrease significantly during the study period (Fig 7D).

### Method of infant delivery

The rate of CDs was relatively stable (p = 0.575) during the study period, but the 31.3% of CD births were associated with almost 40% of the total infant deaths (Table 1). Therefore, CD was associated with increased risk of infant mortality by 51% (95% CI, 1.46–1.57; p<0.001) when compared with vaginal delivery (Table 2). The postneonatal and infant mortality associated with CD linearly decreased significantly during the study period (Fig 7E), but neonatal mortality did not do so.

Both LBW and PTB births are the leading causes of neonatal mortality due to the complications associated with LBW and PTB births. With respect to neonatal mortality rate, LBW and PTB births were more than 62 times (AOR 62.5; 95% CI, 59.4–65.8; p<0.001) and 42 times (AOR 42.6; 95% CI, 40.5–44.8; p<0.001) more likely to result in neonatal deaths compared with those infants who had normal births for LBW and PTB, respectively.

### Leading causes of neonatal, postneonatal, and infant mortality

The most common causes of infant death in the neonatal period are different from those that occur during the postneonatal period. Based on International Statistical Classification of Diseases and Related Health Problems 10th Revision (ICD-10)-WHO Version for 2016 grouping, certain conditions originating in the perinatal period (66.2%) and congenital malformations, deformations and chromosomal abnormalities (27.8%) comprised 94% of the total 13,228 neonatal mortalities during the study period (S5 Table).

As a subgroup in the ICD-10 grouping, the three leading causes of neonatal deaths were disorders related to length of gestation and fetal growth (ICD-10: P05-P08), respiratory and cardiovascular disorders specific to the perinatal period (ICD-10: P20-P29), and fetus and newborn affected by maternal factors and by complications of pregnancy, labor and delivery (ICD-10: P00-P04) (S6 Table). From a clinical perspective, prematurity, congenital heart disease and chromosomal abnormalities are the leading causes of neonatal mortality.

Based on ICD-10, symptoms, signs and abnormal clinical and laboratory findings, not elsewhere classified including sudden infant death syndrome (SIDS) and unknown causes of mortality (28.2%) and congenital malformations, deformations and chromosomal abnormalities (26.8%) comprised 55% of the total 6,073 postneonatal mortalities (S5 Table).

The leading causes of postneonatal deaths were sudden infant death syndrome and unknown causes of mortality (ICD-10: R95-R99) followed by congenital malformations of the circulatory system (ICD-10: Q20-Q28) (S6 Table). From a clinical perspective, SIDS and related unknown causes of mortality followed by congenital heart disease are the leading causes of postneonatal mortality.

Based on ICD-10 codes, certain conditions originating in the perinatal period (47.7%) and congenital malformations, deformations and chromosomal abnormalities (27.5%) comprised 14,503 of the total 19,301 total infant mortalities (S5 Table).

The leading causes of infant mortality were disorders related to length of gestation and fetal growth (ICD-10: P05-P08), respiratory and cardiovascular disorders specific to the perinatal period (ICD-10:P20-P29), fetus and newborn affected by maternal factors and by complications of pregnancy, labor and delivery (ICD-10:P00-P04), sudden infant death syndrome and unknown causes of mortality (ICD-10: R95-R99), and congenital malformations of the circulatory system (ICD-10: Q20-Q28) (S6 Table).

## Discussion

Using birth cohort data from the California Department of Public Health from 2007–2015, we demonstrated a decline in neonatal, postneonatal and infant mortality. In addition, we showed that extremes of maternal age, less than high school education, maternal obesity, low socio-economic status and African-American race were associated with higher IMR. Birth by CD, preterm gestation and SGA/LBW or LGA status were also associated with high IMR.

The rate of preterm birth and evaluation of SGA and LGA in the US depends on the method used to assign gestational age [11]. However, in 2010, Olsen et al. developed and validated new intrauterine growth curves based on a racially diverse US population sample for the identification of SGA and LGA infants [10] and this classification has been employed for a few studies [12, 13]. However, there are remaining gaps in understanding the roles of SGA and LGA in predicting infant mortality rates from population studies using large datasets.

### Infant factors and IMR

The conclusions of this California study support the findings from across the US that the IMR has continued to decline. For the period 2007–2015 in California, IMR dropped, mainly due to changes in infant factors amenable to improvements in medical care, and not in maternal factors that reflect large and widening disparities in sociodemographic and economic characteristics. Infant factors behind the reduction in IMR were both a shift in the distribution of birthweight (higher percentage of AGA births) and a decrease in preterm births leading to a significant reduction in neonatal mortality [1, 18]. Preterm births in the study population decreased from 7.47% in 2007 to 6.89% in 2015, which was consistent with US population data [1]. Improvements in neonatal mortality appear to reflect higher birthweight and fewer preterm births, as well as better neonatal care.

Although there was no significant downward linear trend in the incidence of low birth weight (LBW), there were significant improvements in survival rate for LBW infants, which is only partly explained by a reduction in prematurity. Hence, we speculate that increased access to and advances in neonatal care are significant contributors to decreased IMR. However, while better neonatal care saves lives, it is expensive: the economic impact of this increased survival rate in PTB is enormous, with an average cost of $317,982 for neonatal care for extremely preterm infants (<28 weeks). These costs have been increasing over time [19]. Prematurity and SGA independently influence infant mortality and are important factors influencing infant mortality.

### Sociodemographic factors and IMR

While overall IMR has dropped and infant factors (less prematurity and more AGA births) have improved, significant differences in IMR persist within the population due to disparities in maternal sociodemographic factors, economic status, maternal prepregnancy obesity, and smoking during pregnancy [1]. Our study has established that both maternal prepregnancy obesity and smoking during pregnancy are associated with infant mortality.

### Race and ethnicity

Infants born to African American women had higher IMR, which declined linearly with increasing levels of education. However, the differences in IMRs between infants born to African American women and other ethnic groups persisted. IMRs in infants born to African American women showed a significant increase with a young age at delivery of <20 years. The effects of race and ethnicity on IMRs for Hispanic, Asian, White, and mixed-race women were reduced when the woman had an education level to at least a bachelor’s degree.

### Maternal age

7.8% of the infants born to mothers <20 years of age represented 10.9% of the infant mortality of the study population. Also, this study showed that births at older ages, especially >40 years, were also associated with an increased risk of infant mortality.

These data confirm the potential value of prevention of teenage pregnancy in reducing infant mortality and emphasize a major opportunity for public health intervention. Enhanced family planning programs and easy access to such services may potentially delay the first pregnancies in teenagers until they reach their biological maturity [20].

### Educational attainment

This study showed an increased risk of infant mortality in women with low educational attainment, as almost 59% of the infant deaths were for women who had no more than a high school education and who represented 48.5% of the total births.

Hence, there may be a role for health education to encourage women to stay in school and improve their employment opportunities while advancing both their education and biological maturity. In 2011, Finlay et al. showed that when women delayed pregnancy for a few years to participate in higher education, they became not only better informed, but they had improved job opportunities and healthier lives [20]. These healthier behaviors and attitudes have been shown to be passed on to future offspring, which may then benefit society [20].

### Geographical location

In rural San Joaquin Valley region, women were 51% more likely to experience infant deaths when compared to urban women living in the San Diego area. The IMR is often used as a measure of the overall health of the population of a country, suggesting that similar factors in any geographical area influence population health and infant mortality [20, 21]. Ely et al. also found rural and urban disparities for IMRs in the US [22]. Geographical disparities evident in our data suggest that targeting identified high-risk geographical areas for enhanced antenatal and perinatal services may have a significant beneficial effect on IMR.

### Poverty or economic status

Children of women with characteristics associated with lower economic status, including lower educational attainment, living in rural areas, having Medi-Cal health insurance, and being WIC recipients, were more likely to experience increased IMRs (Tables 1 and 2). While 49.8% of the babies were born to WIC recipients, they represented 60.4% of the total infant mortality. Therefore, there is a clear statistical association between the recipients of WIC benefits and economic status, as an indicator of increased risk for IM.

The WIC setting might provide an opportunity to address risk factors of infant mortality and to carry out interventions such as health education, preventing teen pregnancy, supporting higher education, and providing guidance to get quality prenatal care.

### Maternal overweight and obesity

In 2019, analysis of data from the birth files reported that the rates of pre-pregnancy overweight and obesity were steadily rising in California during the period 2007–2016 [15]. During the study period of 2007–2015, 55% of infant deaths in California were to mothers who were overweight or obese before pregnancy. In the present study, although IMRs significantly declined during the study period 2007–2015, IMRs for infants born to women who were underweight or had class II or class III obesity did not decline significantly. This data strongly suggest that aggressive interventions should be aimed at maintaining appropriate weight at baseline and during pregnancy to minimize infant deaths.

Prenatal care and support from the health education programs may address the maintaining appropriate pre-pregnancy weight to combat infant deaths arising from pre-pregnancy underweight, overweight, and obesity. Prenatal health education would provide the opportunity to address not only poor birth outcomes, including low birth LBW, PTB, and cesarean delivery but also poor health associated with obesity. Diet, lifestyle, and weight have profound and enduring effects on the long-term health of the offspring, and disease risk into adulthood [21]. Therefore, health education for women before and during pregnancy focused specifically at weight maintenance could reduce the national health burden in the 21^st^ century [21].

### Maternal smoking

Smoking during pregnancy (defined here as smoking during both the first and second trimesters), is the most preventable risk factor associated with maternal perinatal behavior. Smoking during pregnancy is associated with a significantly increased risk of adverse birth and maternal outcomes, and differences in rates of LBW, PTB, and SGA between infants of maternal smokers and nonsmokers increased during this period [14]. Smoking cessation is a modifiable behavior likely to contribute to significant reduction infant mortality and represents one of the most useful goals in before-pregnancy and antenatal health counselling of women.

### Health policy targets

This study identified vulnerable or high-risk groups that are special value targets for public efforts to reduce infant mortality. Empowering women, by supporting higher educational needs, also improves socioeconomic status and employment opportunities, which are major indicators of health disparities. Increased educational attainment may modify adverse health behaviors that include teen pregnancy, smoking, substance and alcohol abuse during pregnancy.

We report a distinct difference in the etiology of neonatal and postneonatal mortality. The leading causes of neonatal deaths were due to disorders related to length of gestation and fetal growth, respiratory and cardiovascular disorders specific to the perinatal period, and fetus and newborn affected by maternal factors and by complications of pregnancy, labor and delivery. The leading causes of postneonatal deaths were sudden infant death syndrome, followed by congenital malformations.

Based on these findings, we speculate that public education focusing on maternal obesity and smoking are likely to impact all aspects of infant mortality–however, we anticipate that the impact is likely to be more profound with neonatal rather than postneonatal mortality. On the other hand, educational campaigns focusing on “back-to-sleep” to reduce SIDS are likely to have an impact on postneonatal mortality.

### Caesarean Delivery (CD)

Another predictor unveiled in our data is the effect of CD on IMR. Molina et al. indicated that the optimal CD rate was approximately 19 cesarean deliveries per 100 live births [23]. However, our cohort had a CD rate of 31.3%, which was associated with almost 40% of infant mortality. In other words, almost 40% of the infants who died before 1 year of age were delivered by caesarean section. Therefore, reducing the CD rate may potentially reduce IMR.

### Study strengths and limitations

This study has several significant strengths. First, we used a large study sample size of more than 4.5 million singleton live births that included all births in California during the period 2007–2015. Second, the population studied is ethnically, geographically, and socioeconomically diverse. Third, we employed several predictors not previously evaluated to assess high-risk maternal characteristic, such as obesity. The body mass index (BMI) of women was recorded, and included as a reflection of health of women during pregnancy. Hence this report affirms the adverse effects of excessive weight gain on IMR and adds this adverse characteristics as a significant but also modifiable factor in infant health. Both smoking and obesity were included in the data analysis as covariates and are both key predictors for IMR. Previous population studies on IMRs did not analyze these datasets.

Our study has several limitations. This retrospective study analyzed California vital statistics data, and recent reports have supported the value and quality of this database for epidemiological studies on maternal and neonatal health [21–23]. However, the California vital statistics data may not include all potential confounding variables for the assessment of IMRs. For example, the influence of parental and family relationships, maternal employment status, and other factors associated with socioeconomic inequalities. In addition, some maternal data, including pre-pregnancy weight and BMI, and smoking history, were either self-reported or abstracted from the medical records, and the possibility of incorrect data for these factors cannot be excluded. Analysis and interpretation of risk profiles for IMRs are complex and include aspects of maternal lifestyle and health behaviors that are difficult to quantify and to modify [24]. Because this retrospective study included the analysis of anonymized patient data, the statistical analysis, individual pregnancies could not be linked to the same mother. Therefore, some women might have been analyzed more than once, as the study was undertaken on risk factors associated with individual pregnancies, and not on individual women. Finally, the evaluation of SGA and LGA depends on the method used to assign gestational age [19]. We employed the intrauterine growth curves developed and validated by Olsen et al. in 2010 based on a racially diverse sample of the US population [20].

## Conclusions

This study has highlighted overall improvements in IMRs in California, with declines in almost every category of risk. However, there are several troublesome findings: first, our data has highlighted persistent and increasing disparities in the IMR depending on maternal sociodemographic and economic factors. Sociodemographic factors associated with higher infant deaths included a low level of maternal education, very young and older maternal age, African American race, and rural residence. Lower economic status likewise had a strong association with higher infant deaths.

Second, both smoking and obesity during pregnancy, appear to be exerting an increasing detrimental effect on infant mortality. Our data specifically highlights the previously poorly-recognized association of maternal obesity with higher IMR.

Our report elucidates a substantial worsening of disparities in maternal sociodemographic and economic characteristic as well as prepregnancy obesity and smoking during pregnancy continue to contribute significantly to IMR. In contrast, we have also uncovered unequivocal general improvements in infant predictors of IMR. These improvements were associated with higher birthweights and fewer preterm births, as well as better neonatal care. Further efforts to reduce the incidence of or mitigate the impact of preterm birth, low birth weight, and congenital malformations may spur further improvements overall and reduce disparities.

## Supporting information

S1 Fig Unadjusted SGA (small-for-gestational-age) births (%) in California for singleton births only for the period 2007–2015 by (A) maternal age and maternal race and ethnicity, and (B) maternal education and maternal race and ethnicity.
(TIF)

S2 Fig Unadjusted LGA (large-for-gestational-age) births (%) in California for singleton births only for the period 2007–2015 by (A) maternal age and maternal race and ethnicity, and (B) maternal education and maternal race and ethnicity.
(TIF)

S1 Table Infant mortality, neonatal mortality, and postneonatal mortality rates (per 1,000 live singleton births), mean age of women (years), mean birth weight (grams), mean gestational age (weeks), LBW (%), PTB (%), (LBW + PTB) (%), Cesarean delivery (%) (for study population B), and SGA (%), AGA (%), and LGA (%) (for study subpopulation B) for 2007 to 2015. Abbreviations: LBW, low birth weight; PTB, preterm birth; SGA, small-for-gestational-age; AGA, appropriate-for-gestational-age; LGA, large-for-gestational-age. Study Population A was defined in Fig 1 and Study Subpopulation A was defined in Fig 2.
(DOCX)

S2 Table Comparison of maternal race and ethnic groups within maternal education groups for Infant Mortality Rates (IMRs). Mean IMRs (infant mortality rates) in the same column with different superscripts significantly differ (p<0.05). MSD = Minimum significance difference at p = 0.05. ^a^ p value for Analysis of Variance test.
(DOCX)

S3 Table Crude and adjusted odds ratios (with 95% confidence intervals in parentheses) for SGA births for maternal characteristics in California for the period 2007–2015. OR: odds ratio; AOR: adjusted odds ratio; CI: confidence interval. ^a^ p value for χ^2^ test. ^b^ Ref = Reference group. ^***c***^ Women, Infants, and Children program. All live singleton births as defined in Study Subpopulation A in Fig 2.
(DOCX)

S4 Table Crude and adjusted odds ratios (with 95% confidence intervals in parentheses) for LGA births for maternal characteristics in California for the period 2007–2015. OR: odds ratio; AOR: adjusted odds ratio; CI: confidence interval. ^a^ p value for χ^2^ test. ^b^ Ref = Reference group; All live singleton births as defined in Study Subpopulation A in Fig 2.
(DOCX)

S5 Table Causes of neonatal, postneonatal, and infant deaths by main ICD-10 ^a^ groups during 2007 through 2015. ^a^ International Statistical Classification of Diseases and Related Health Problems 10th Revision (ICD-10)-WHO Version for 2016 https://icd.who.int/browse10/2016/en#/VII.
(DOCX)

S6 Table Leading causes of neonatal, postneonatal, and infant deaths by ICD-10 subgroups during 2007 through 2015. (DOCX)
