# Supplementary figures and images for: Maternal and infant predictors of infant mortality in California, 2007–2015
Source: PLoS One. 2020 Aug 6;15(8):e0236877. doi: 10.1371/journal.pone.0236877 (PMC7410301; doi:10.1371/journal.pone.0236877)

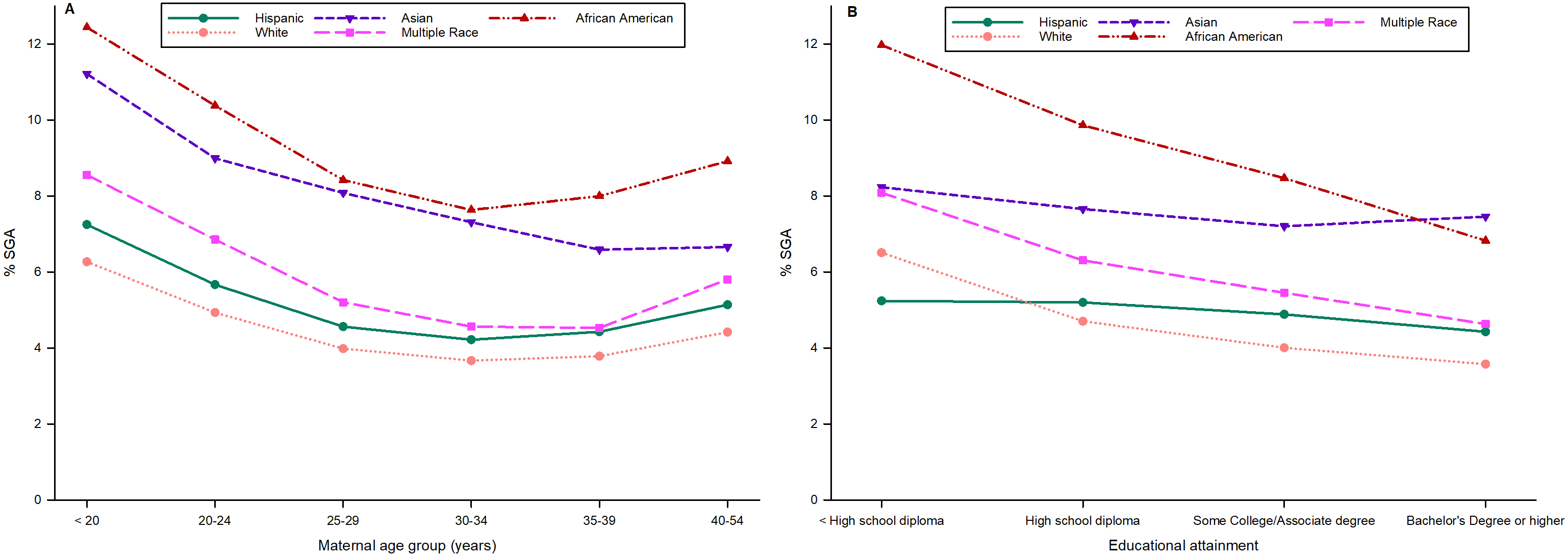

Supplement: S1 Fig — Unadjusted SGA (small-for-gestational-age) births (%) in California for singleton births only for the period 2007–2015 by (A) maternal age and maternal race and ethnicity, and (B) maternal education and maternal race and ethnicity. (TIF) [file pone.0236877.s001.tif]

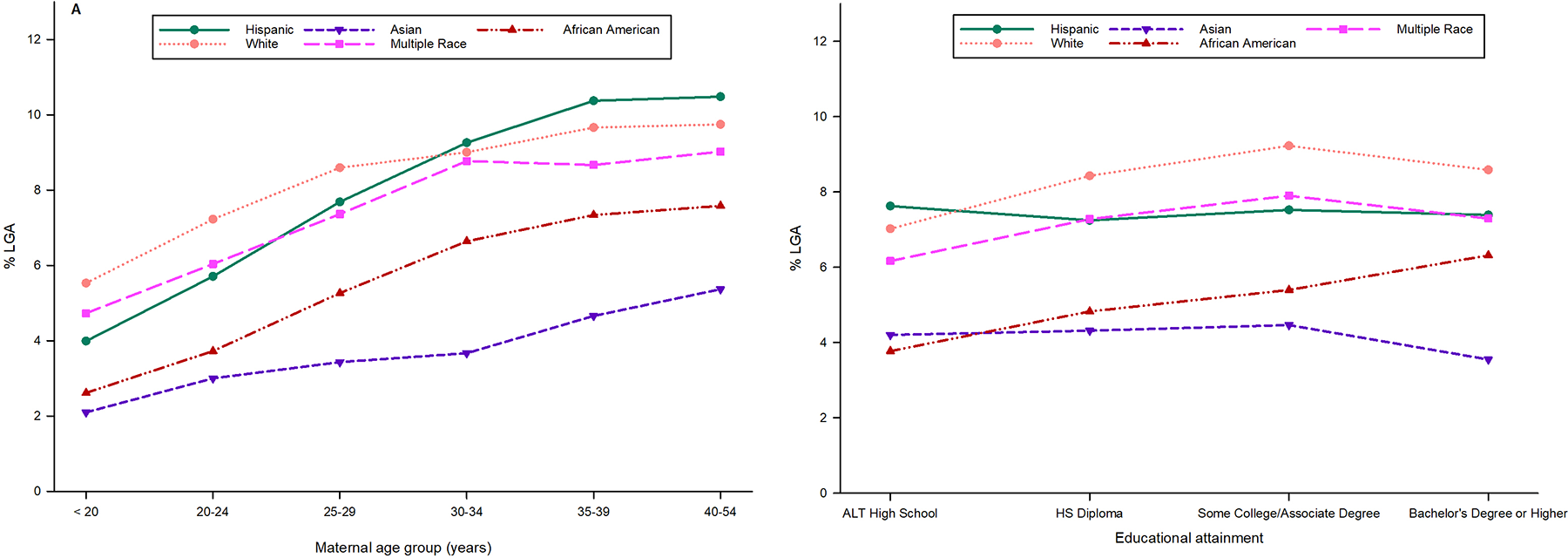

Supplement: S2 Fig — Unadjusted LGA (large-for-gestational-age) births (%) in California for singleton births only for the period 2007–2015 by (A) maternal age and maternal race and ethnicity, and (B) maternal education and maternal race and ethnicity. (TIF) [file pone.0236877.s002.tif]
