# Supplementary material for: Maternal and infant predictors of infant mortality in California, 2007–2015
Source: PLoS One. 2020 Aug 6;15(8):e0236877. doi: 10.1371/journal.pone.0236877 (PMC7410301; doi:10.1371/journal.pone.0236877)
Supplement: S1 Table — Abbreviations: LBW, low birth weight; PTB, preterm birth; SGA, small-for-gestational-age; AGA, appropriate-for-gestational-age; LGA, large-for-gestational-age. Study Population A was defined in Fig 1 and Study Subpopulation A was defined in Fig 2. (DOCX) [file pone.0236877.s003.docx]

**Supplementary Table 1:** Infant mortality, neonatal mortality, and postneonatal mortality rates (per 1,000 live singleton births), mean age of women (years), mean birth weight (grams), mean gestational age (weeks), LBW (%), PTB (%), (LBW + PTB) (%), Cesarean Delivery (%) (for Study Population B), and SGA (%), AGA (%), and LGA (%) (for Study Subpopulation B) for 2007 to 2015.

| **Year** | **Infant mortality rate (per 1,000 live singleton births)** | **Neonatal mortality rate (per 1,000 live singleton births)** | **Postneonatal mortality rate (per 1,000 live singleton births)** | **Mean age of women (years)** |  | **Mean birth weight (grams)** | **Mean gestational age (weeks)** |  | **LBW**  **(%)** | **PTB**  **(%)** | **(LBW + PTB)**  **(%)** | **Cesarean Delivery**  **(%)** | **SGA**  **(%)** | **AGA**  **(%)** | **LGA**  **(%)** |
| --- | --- | --- | --- | --- | --- | --- | --- | --- | --- | --- | --- | --- | --- | --- | --- |
| 2007 | 4.68 | 3.15 | 1.53 | 27.94 |  | 3,333 | 37.94 |  | 5.27 | 7.47 | 3.41 | 30.52 | 5.31 | 86.81 | 7.88 |
| 2008 | 4.68 | 3.15 | 1.53 | 28.01 |  | 3,332 | 38.34 |  | 5.22 | 7.41 | 3.43 | 31.03 | 5.26 | 86.99 | 7.75 |
| 2009 | 4.36 | 2.93 | 1.43 | 28.13 |  | 3,329 | 38.54 |  | 5.19 | 7.10 | 3.40 | 31.43 | 5.38 | 87.25 | 7.37 |
| 2010 | 4.30 | 2.94 | 1.36 | 28.30 |  | 3,329 | 38.63 |  | 5.25 | 6.97 | 3.43 | 31.50 | 5.45 | 87.26 | 7.29 |
| 2011 | 4.36 | 3.03 | 1.33 | 28.51 |  | 3,330 | 38.65 |  | 5.18 | 6.88 | 3.41 | 31.66 | 5.38 | 87.44 | 7.17 |
| 2012 | 4.04 | 2.76 | 1.28 | 28.70 |  | 3,331 | 38.65 |  | 5.17 | 6.90 | 3.42 | 31.72 | 5.31 | 87.44 | 7.25 |
| 2013 | 4.19 | 2.90 | 1.30 | 28.92 |  | 3,327 | 38.68 |  | 5.26 | 6.84 | 3.47 | 31.67 | 5.42 | 87.59 | 6.99 |
| 2014 | 3.96 | 2.81 | 1.15 | 29.16 |  | 3,328 | 38.68 |  | 5.12 | 6.70 | 3.34 | 31.13 | 5.47 | 87.58 | 6.95 |
| 2015 | 3.90 | 2.71 | 1.19 | 29.35 |  | 3,321 | 38.68 |  | 5.29 | 6.89 | 3.49 | 30.78 | 5.41 | 87.82 | 6.77 |
| P value for linear regression | < .001 | 0.002 | < .001 | <.0001 |  | 0.013 | 0.012 |  | 0.737 | 0.002 | 0.620 | 0.575 | 0.069 | <.0001 | <.0001 |

Abbreviations: LBW, low birth weight; PTB, preterm birth; SGA, small-for-gestational-age; AGA, appropriate-for-gestational-age; LGA, large-for-gestational-age

Cochran-Armitage trend test also showed the declining trends in neonatal (p <0.001), postneonatal (p<0.001), and infant mortality rates (p<0.001) for singleton births from 2007 to 2015.

Study Population A was defined in Fig. 1 and Study Subpopulation A was defined in Fig. 2
