# Supplementary material for: Maternal and infant predictors of infant mortality in California, 2007–2015
Source: PLoS One. 2020 Aug 6;15(8):e0236877. doi: 10.1371/journal.pone.0236877 (PMC7410301; doi:10.1371/journal.pone.0236877)
Supplement: S2 Table — Mean IMRs (infant mortality rates) in the same column with different superscripts significantly differ (p<0.05). MSD = Minimum significance difference at p = 0.05. a p value for Analysis of Variance test. (DOCX) [file pone.0236877.s004.docx]

**Supplementary Table 2.** Comparison of maternal race and ethnic groups within maternal education groups for Infant Mortality Rates (IMRs).

| Maternal education group | | | | | | | | | | | |
| --- | --- | --- | --- | --- | --- | --- | --- | --- | --- | --- | --- |
| < High school | |  | High school diploma | |  | Some college or associate degree | |  | Bachelor's degree or higher | |  |
| Race and ethnicity | Mean IMR |  | Race and ethnicity | Mean IMR |  | Race and ethnicity | Mean IMR |  | Race and ethnicity | Mean IMR |  |
|  |  |  |  |  |  |  |  |  |  |  |  |
| **African American** | **10.70^b^** |  | **African American** | **9.72^b^** |  | **African American** | **8.14^b^** |  | **African American** | **5.69^b^** |  |
| **White** | **7.01^c^** |  | **White** | **4.47^c^** |  | Hispanic | 3.77^c^ |  | Hispanic | 2.60^c^ |  |
| Asian | 5.27^d^ |  | Asian | 4.27^c^ |  | **White** | **3.74^c^** |  | Asian | 2.43^c^ |  |
| Hispanic | 4.59^d^ |  | Hispanic | 4.21^c^ |  | Asian | 3.71^c^ |  | **White** | **2.01^c^** |  |
| p value ^a^ | <.001 |  |  | <.001 |  |  | <.001 |  |  | <.001 |  |
| MSD | 1.567 |  |  | 1.008 |  |  | 0.524 |  |  | 1.318 |  |

Mean IMRs (infant mortality rates) in the same column with different superscripts significantly differ (p<0.05)

MSD = Minimum significance difference at p=0.05

^a^ p value for Analysis of Variance test
