# Supplementary material for: Maternal and infant predictors of infant mortality in California, 2007–2015
Source: PLoS One. 2020 Aug 6;15(8):e0236877. doi: 10.1371/journal.pone.0236877 (PMC7410301; doi:10.1371/journal.pone.0236877)
Supplement: S3 Table — OR: odds ratio; AOR: adjusted odds ratio; CI: confidence interval. a p value for χ2 test. b Ref = Reference group. c Women, Infants, and Children program. All live singleton births as defined in Study Subpopulation A in Fig 2. (DOCX) [file pone.0236877.s005.docx]

**Supplementary Table 3.** Crude and adjusted odds ratios (with 95% confidence intervals in parentheses) for SGA births for maternal characteristics in California for the period 2007–2015.

| **Characteristic** | **Crude odds ratio** | |  | **Adjusted odds ratio** | |
| --- | --- | --- | --- | --- | --- |
|  | **OR (95% CI)** | **p value^a^** |  | **AOR (95% CI)** | **p value^a^** |
| ***Birth year*** |  |  |  |  |  |
| 2008 | 0.99 (0.97–1.01) | 0.272 |  | 1.00 (0.98–1.02) | 0.689 |
| 2009 | 1.02 (1.00–1.04) | 0.049 |  | 1.03 (1.01–1.05) | 0.006 |
| 2010 | 1.04 (1.02–1.06) | <.001 |  | 1.05 (1.03–1.07) | <.001 |
| 2011 | 1.03 (1.01–1.04) | 0.007 |  | 1.06 (1.04–1.08) | <.001 |
| 2012 | 1.01 (0.99–1.03) | 0.182 |  | 1.03 (1.01–1.05) | 0.001 |
| 2013 | 1.04 (1.02–1.06) | <.001 |  | 1.07 (1.05–1.09) | <.001 |
| 2014 | 1.04 (1.02–1.06) | <.001 |  | 1.09 (1.07–1.11) | <.001 |
| 2015 | 1.03 (1.01–1.05) | 0.001 |  | 1.09 (1.07–1.11) | <.001 |
| 2007 (ref) | Ref^b^ |  |  | Ref^b^ |  |
| ***Maternal age (years)*** |  |  |  |  |  |
| < 20 | 0.78 (0.77–0.79) | <.001 |  | 1.03 (1.01–1.05) | 0.001 |
| 20–24 | 0.64 (0.63–0.65) | <.001 |  | 1.02 (1.00–1.04) | 0.124 |
| 25–29 | 0.61 (0.60–0.62) | <.001 |  | 1.05 (1.02–1.07) | <.001 |
| 35–39 | 0.61 (0.60–0.62) | <.001 |  | 1.12 (1.09–1.14) | <.001 |
| 40–54 | 0.68 (0.67–0.70) | <.001 |  | 1.26 (1.22–1.30) | <.001 |
| 30-34 (ref) | Ref^b^ |  |  | Ref^b^ |  |
| ***Maternal race/ethnicity*** |  |  |  |  |  |
| African American | 2.39 (2.35–2.43) | <.001 |  | 2.29 (2.25–2.34) | <.001 |
| American Indian | 1.25 (1.16–1.34) | <.001 |  | 1.23 (1.13–1.34) | <.001 |
| Asian | 1.89 (1.86–1.92) | <.001 |  | 1.94 (1.91–1.97) | <.001 |
| Hispanic | 1.26 (1.24–1.27) | <.001 |  | 1.25 (1.23–1.27) | <.001 |
| Multiple Race | 1.40 (1.36–1.45) | <.001 |  | 1.35 (1.31–1.40) | <.001 |
| Pacific Islander | 1.14 (1.06–1.22) | <.001 |  | 1.31 (1.21–1.41) | <.001 |
| White (ref) | Ref^b^ |  |  | Ref^b^ |  |
| ***Maternal education*** |  |  |  |  |  |
| < High school | 1.14 (1.12–1.15) | <.001 |  | 1.24 (1.22–1.26) | <.001 |
| High school diploma | 1.12 (1.11–1.13) | <.001 |  | 1.17 (1.15–1.19) | <.001 |
| Some college/associate degree | 1.03 (1.01–1.04) | <.001 |  | 1.09 (1.07–1.11) | <.001 |
| Bachelor's degree or higher (ref) | Ref^b^ |  |  | Ref^b^ |  |
| ***Maternal nativity*** |  |  |  |  |  |
| United States–born | 0.94 (0.93–0.94) | <.001 |  | 1.03 (1.02–1.04) | <.001 |
| Foreign–born (ref) | Ref^b^ |  |  | Ref^b^ |  |
| ***Maternal demographic region*** | |  |  |  |  |
| Central Coast | 0.98 (0.96–1.01) | 0.117 |  | 1.05 (1.03–1.08) | <.001 |
| Greater Bay Area | 1.13 (1.11–1.15) | <.001 |  | 1.05 (1.03–1.07) | <.001 |
| Inland Empire | 1.08 (1.06–1.10) | <.001 |  | 1.07 (1.05–1.10) | <.001 |
| Los Angeles County | 1.20 (1.18–1.22) | <.001 |  | 1.13 (1.11–1.16) | <.001 |
| Northern and Sierra | 1.04 (1.01–1.07) | 0.005 |  | 1.05 (1.02–1.09) | 0.002 |
| Orange County | 1.06 (1.03–1.08) | <.001 |  | 1.05 (1.02–1.07) | 0.000 |
| Sacramento Area | 1.02 (1.00–1.05) | 0.090 |  | 1.01 (0.98–1.03) | 0.716 |
| San Joaquin Valley | 1.12 (1.10–1.14) | <.001 |  | 1.11 (1.09–1.14) | <.001 |
| San Diego Area (ref) | Ref^b^ |  |  | Ref^b^ |  |
| ***Source of prenatal care payment*** | |  |  |  |  |
| Medi–Cal (Public) | 1.20 (1.19–1.21) | <.001 |  | 1.15 (1.13–1.16) | <.001 |
| Private insurance (ref) | Ref^b^ |  |  | Ref^b^ |  |
| ***WIC^c^ participation*** |  |  |  |  |  |
| No | 0.87 (0.87–0.88) | <.001 |  | 1.03 (1.01–1.04) | 0.000 |
| Yes (ref) | Ref^b^ |  |  | Ref^b^ |  |
| ***First trimester prenatal care initiation*** | |  |  |  |  |
| No | 1.21 (1.19–1.22) | <.001 |  | 1.10 (1.09–1.12) | <.001 |
| Yes (ref) | Ref^b^ |  |  | Ref^b^ |  |
| ***Parity*** |  |  |  |  |  |
| Primiparous | 1.62 (1.56–1.67) | <.001 |  | 1.95 (1.87–2.03) | <.001 |
| Multiparous 2–5 | 0.88 (0.85–0.91) | <.001 |  | 1.05 (1.01–1.09) | 0.024 |
| Multiparous 6–12 (ref) | Ref^b^ |  |  | Ref^b^ |  |
| ***Maternal smoking during both first and second trimesters*** | | |  |  |  |
| Yes | 1.96 (1.91–2.02) | <.001 |  | 2.10 (2.03–2.16) | <.001 |
| No (ref) | Ref^b^ |  |  | Ref^b^ |  |
| ***Maternal prepregnancy body mass index (kg/m^2^)*** | | |  |  |  |
| Underweight, <18.5 | 1.70 (1.67–1.73) | <.001 |  | 1.51 (1.48–1.54) | <.001 |
| Overweight, 25.0–29.9 | 0.79 (0.78–0.79) | <.001 |  | 0.82 (0.81–0.83) | <.001 |
| Obese I–30.0–34.9 | 0.72 (0.71–0.73) | <.001 |  | 0.76 (0.75–0.77) | <.001 |
| Obese II, 35.0–39.9 | 0.68 (0.66–0.69) | <.001 |  | 0.71 (0.69–0.73) | <.001 |
| Obese III, ≥ 40 | 0.65 (0.63–0.67) | <.001 |  | 0.68 (0.66–0.70) | <.001 |
| Normal, 18.5–24.9 (ref) | Ref^b^ |  |  | Ref^b^ |  |

OR: odds ratio; AOR: adjusted odds ratio; CI: confidence interval

^a^ p value for χ^2^ test

^b^ Ref = Reference group

***^c^*** Women, Infants, and Children program

All live singleton births as defined in Study Subpopulation A in Fig. 2
