# Supplementary material for: Maternal and infant predictors of infant mortality in California, 2007–2015
Source: PLoS One. 2020 Aug 6;15(8):e0236877. doi: 10.1371/journal.pone.0236877 (PMC7410301; doi:10.1371/journal.pone.0236877)
Supplement: S4 Table — OR: odds ratio; AOR: adjusted odds ratio; CI: confidence interval. a p value for χ2 test. b Ref = Reference group; All live singleton births as defined in Study Subpopulation A in Fig 2. (DOCX) [file pone.0236877.s006.docx]

**Supplementary Table 4.** Crude and adjusted odds ratios (with 95% confidence intervals in parentheses) for LGA births for maternal characteristics in California for the period 2007–2015.

| **Characteristic** | **Crude odds ratio** | |  | **Adjusted odds ratio** | |
| --- | --- | --- | --- | --- | --- |
|  | **OR (95% CI)** | **p value^a^** |  | **AOR (95% CI)** | **p value^a^** |
| ***Birth year*** |  |  |  |  |  |
| 2008 | 0.98 (0.96–0.99) | <.001 |  | 0.97 (0.96–0.99) | <.001 |
| 2009 | 0.92 (0.91–0.93) | <.001 |  | 0.90 (0.89–0.92) | <.001 |
| 2010 | 0.91 (0.90–0.93) | <.001 |  | 0.89 (0.87–0.90) | <.001 |
| 2011 | 0.90 (0.88–0.91) | <.001 |  | 0.86 (0.85–0.87) | <.001 |
| 2012 | 0.91 (0.89–0.92) | <.001 |  | 0.87 (0.85–0.88) | <.001 |
| 2013 | 0.87 (0.86–0.89) | <.001 |  | 0.83 (0.81–0.84) | <.001 |
| 2014 | 0.87 (0.86–0.88) | <.001 |  | 0.82 (0.81–0.83) | <.001 |
| 2015 | 0.85 (0.83–0.86) | <.001 |  | 0.79 (0.77–0.80) | <.001 |
| 2007 (ref) | Ref^b^ |  |  | Ref^b^ |  |
| ***Maternal age (years)*** |  |  |  |  |  |
| 20-24 Years | 1.45 (1.42–1.48) | <.001 |  | 1.10 (1.08–1.13) | <.001 |
| 25-29 Years | 1.87 (1.83–1.91) | <.001 |  | 1.30 (1.27–1.33) | <.001 |
| 30-34 Years | 2.05 (2.01–2.09) | <.001 |  | 1.44 (1.41–1.47) | <.001 |
| 35-39 Years | 2.27 (2.23–2.32) | <.001 |  | 1.56 (1.52–1.60) | <.001 |
| 40-54 Years | 2.36 (2.31–2.42) | <.001 |  | 1.57 (1.52–1.61) | <.001 |
| 30-34 (ref) | Ref^b^ |  |  | Ref^b^ |  |
| ***Maternal race/ethnicity*** |  |  |  |  |  |
| African American | 1.33 (1.30–1.37) | <.001 |  | 1.10 (1.07–1.14) | <.001 |
| American Indian | 2.83 (2.68–3.00) | <.001 |  | 2.16 (2.03–2.30) | <.001 |
| Hispanic | 2.00 (1.97–2.03) | <.001 |  | 1.67 (1.64–1.70) | <.001 |
| Multiple Race | 1.99 (1.93–2.05) | <.001 |  | 1.78 (1.73–1.84) | <.001 |
| Pacific Islander | 3.74 (3.57–3.92) | <.001 |  | 2.49 (2.37–2.62) | <.001 |
| White | 2.35 (2.32–2.39) | <.001 |  | 2.11 (2.07–2.15) | <.001 |
| Asian (ref) | Ref^b^ |  |  | Ref^b^ |  |
| ***Maternal education*** |  |  |  |  |  |
| < High school | 1.10 (1.08–1.11) | <.001 |  | 0.99 (0.97–1.00) | 0.081 |
| High school diploma | 1.07 (1.06–1.08) | <.001 |  | 0.99 (0.98–1.01) | 0.284 |
| Some college/associate degree | 1.14 (1.13–1.15) | <.001 |  | 1.01 (1.00–1.02) | 0.127 |
| Bachelor's degree or higher (ref) | Ref^b^ |  |  | Ref^b^ |  |
| ***Maternal nativity*** |  |  |  |  |  |
| Foreign-born | 0.89 (0.89–0.90) | <.001 |  | 1.02 (1.01–1.03) | <.001 |
| United States–born (ref) | Ref^b^ |  |  | Ref^b^ |  |
| ***Maternal demographic region*** | |  |  |  |  |
| Central Coast | 1.21 (1.19–1.23) | <.001 |  | 1.10 (1.08–1.12) | <.001 |
| Greater Bay Area | 1.11 (1.09–1.12) | <.001 |  | 1.14 (1.12–1.15) | <.001 |
| Inland Empire | 1.10 (1.09–1.11) | <.001 |  | 1.02 (1.01–1.03) | <.001 |
| Northern and Sierra | 1.32 (1.30–1.35) | <.001 |  | 1.17 (1.14–1.19) | <.001 |
| Orange County | 1.06 (1.04–1.08) | <.001 |  | 1.06 (1.04–1.08) | <.001 |
| Sacramento Area | 1.33 (1.31–1.35) | <.001 |  | 1.24 (1.22–1.26) | <.001 |
| San Diego Area | 1.17 (1.16–1.19) | <.001 |  | 1.14 (1.12–1.16) | <.001 |
| San Joaquin Valley | 1.21 (1.19–1.22) | <.001 |  | 1.09 (1.08–1.11) | <.001 |
| Los Angeles County (ref) | Ref^b^ |  |  | Ref^b^ |  |
| ***Source of prenatal care payment*** | |  |  |  |  |
| Private | 1.06 (1.05–1.07) | <.001 |  | 1.06 (1.05–1.07) | <.001 |
| Medi–Cal (Public) (ref) | Ref^b^ |  |  | Ref^b^ |  |
| ***First trimester prenatal care initiation*** | |  |  |  |  |
| Yes | 1.08 (1.07–1.09) | <.001 |  | 1.08 (1.06–1.09) | <.001 |
| No (ref) | Ref^b^ |  |  | Ref^b^ |  |
| ***Parity*** |  |  |  |  |  |
| Multiparous 2-5 | 1.76 (1.74–1.77) | <.001 |  | 1.47 (1.46–1.49) | <.001 |
| Multiparous 6-12 | 2.53 (2.47–2.59) | <.001 |  | 1.78 (1.73–1.83) | <.001 |
| Primiparous | Ref^b^ |  |  | Ref^b^ |  |
| ***Maternal smoking during both first and second trimesters*** | | |  |  |  |
| No | 1.31 (1.27–1.35) | <.001 |  | 1.63 (1.57–1.70) | <.001 |
| Yes (ref) | Ref^b^ |  |  | Ref^b^ |  |
| ***Maternal prepregnancy body mass index (kg/m^2^)*** | | | | |  |
| Normal, 18.5-24.9 | 2.03 (1.97–2.10) | <.001 |  | 1.80 (1.74–1.87) | <.001 |
| Overweight, 25.0-29.9 | 3.41 (3.31–3.52) | <.001 |  | 2.91 (2.81–3.02) | <.001 |
| Obese I, 30.0-34.9 | 4.60 (4.46–4.75) | <.001 |  | 3.89 (3.76–4.03) | <.001 |
| Obese II, 35.0-39.9 | 5.84 (5.65–6.04) | <.001 |  | 4.90 (4.72–5.08) | <.001 |
| Obese III, ≥ 40 | 7.12 (6.88–7.37) | <.001 |  | 5.95 (5.73–6.18) | <.001 |
| Underweight, <18.5 (ref) | Ref^b^ |  |  | Ref^b^ |  |

OR: odds ratio; AOR: adjusted odds ratio; CI: confidence interval

^a^ p value for χ^2^ test

^b^ Ref = Reference group; All live singleton births as defined in Study Subpopulation A in Fig. 2
