# Supplementary material for: Maternal and infant predictors of infant mortality in California, 2007–2015
Source: PLoS One. 2020 Aug 6;15(8):e0236877. doi: 10.1371/journal.pone.0236877 (PMC7410301; doi:10.1371/journal.pone.0236877)
Supplement: S5 Table — a International Statistical Classification of Diseases and Related Health Problems 10th Revision (ICD-10)-WHO Version for 2016 https://icd.who.int/browse10/2016/en#/VII. (DOCX) [file pone.0236877.s007.docx]

**Supplementary Table 5:** Causes of neonatal, postneonatal, and infant deaths by main ICD-10^a^ groups during 2007 through 2015

| ICD-10 ^a^ | Description | Neonatal mortality | Postneonatal mortality | Infant mortality |
| --- | --- | --- | --- | --- |
| A00-B99 | Certain infectious and parasitic diseases | 31 (0.2) | 325 (5.4) | 356 (1.8) |
| C00-D48 | Neoplasms | 36 (0.3) | 93 (1.5) | 129 (0.7) |
| D50-D89 | Diseases of the blood and blood-forming organs and certain disorders involving the immune mechanism | 43 (0.3) | 50 (0.8) | 93 (0.5) |
| E00-E90 | Endocrine, nutritional and metabolic diseases | 70 (0.5) | 111 (1.8) | 181 (0.9) |
| G00-G99 | Diseases of the nervous system | 59 (0.4) | 246 (4.1) | 305 (1.6) |
| H60-H95 | Diseases of the ear and mastoid process | 0 | 5 (0.1) | 5 (0.0) |
| I00-I99 | Diseases of the circulatory system | 81 (0.6) | 292 (4.8) | 373 (1.9) |
| J00-J99 | Diseases of the respiratory system | 13 (0.1) | 343 (5.6) | 356 (1.8) |
| K00-K93 | Diseases of the digestive system | 39 (0.3) | 123 (2.0) | 162 (0.8) |
| L00-L99 | Diseases of the skin and subcutaneous tissue | 0 (0.0) | 1 (0.0) | 1 (0.0) |
| M00-M99 | Diseases of the musculoskeletal system and connective tissue | 5 (0.0) | 4 (0.1) | 9 (0.0) |
| N00-N99 | Diseases of the genitourinary system | 25 (0.2) | 28 (0.5) | 53 (0.3) |
| P00-P96 | Certain conditions originating in the perinatal period | 8,761 (66.2) | 437 (7.2) | 9,198 (47.7) |
| Q00-Q99 | Congenital malformations, deformations and chromosomal abnormalities | 3,677 (27.8) | 1,628 (26.8) | 5,305 (27.5) |
| R00-R99 | Symptoms, signs and abnormal clinical and laboratory findings, not elsewhere classified | 281 (2.1) | 1,711 (28.2) | 1,992 (10.3) |
| V01-Y98 | External causes of morbidity and mortality | 102 (0.8) | 673 (11.1) | 775 (4.0) |
| Z00-Z99 | Factors influencing health status and contact with health services | 5 (0.0) | 3 (0.0) | 8 (0.0) |
|  | Total | 13,228 (68.5%) | 6,073  (31.5%) | 19,301 (100%) |

^a^ International Statistical Classification of Diseases and Related Health Problems 10th Revision (ICD-10)-WHO Version for 2016 https://icd.who.int/browse10/2016/en#/VII
