# Supplementary material for: Maternal and infant predictors of infant mortality in California, 2007–2015
Source: PLoS One. 2020 Aug 6;15(8):e0236877. doi: 10.1371/journal.pone.0236877 (PMC7410301; doi:10.1371/journal.pone.0236877)
Supplement: S6 Table — (DOCX) [file pone.0236877.s008.docx]

**Supplementary Table 6:** Leading causes of neonatal, postneonatal, and infant deaths by ICD-10 subgroups during 2007 through 2015

| Group | ICD-10^a^ | ICD-10 Description | Neonatal mortality | Postneonatal mortality | Infant mortality |
| --- | --- | --- | --- | --- | --- |
| 1 | P05-P08 | Disorders related to length of gestation and fetal growth | 2,683 (20.3) | 45 (0.7) | 2,728 (14.1) |
| *1.A* | *P07.2* | *Extreme immaturity Less than 28 completed weeks (less than 196 completed days) of gestation.* | *2302 (17.4)* | *22 (0.4)* | *2324 (12.0)* |
| *1.B* | *P07.3* | *Other preterm infants 28 completed weeks or more but less than 37 completed weeks (196 completed days but less than 259 completed days) of gestation.* | *319 (2.4)* | *12 (0.2)* | *331 (1.7)* |
| *1.C* | *P05.9* | *Slow fetal growth, unspecified Fetal growth retardation NOS* | *49 (0.4)* | *10 (0.2)* | *59 (0.3)* |
|  |  |  |  |  |  |
| 2 | P20-P29 | Respiratory and cardiovascular disorders specific to the perinatal period | 2,122 (16.0) | 178 (2.9) | 2,300 (11.9) |
| *2.A* | *P29.1* | *Neonatal cardiac dysrhythmia* | *602 (4.6)* | *3 (0.0)* | *605 (3.1)* |
| *2.B* | *P29.0* | *Neonatal cardiac failure* | *336 (2.5)* | *5 (0.1)* | *341 (1.8)* |
| *2.C* | *P22.0* | *Respiratory distress syndrome of newborn* | *271 (2.0)* | *12 (0.2)* | *283 (1.5)* |
|  |  |  |  |  |  |
| 3 | P00-P04 | Fetus and newborn affected by maternal factors and by complications of pregnancy, labor and delivery | 2,247 (17.0) | 45 (0.7) | 2,292 (11.9) |
| *3.A* | *P01.1* | *Fetus and newborn affected by premature rupture of membranes* | *633 (4.8)* | *6 (0.1)* | *639 (3.3)* |
| *3.B* | *P01.0* | *Fetus and newborn affected by incompetent cervix* | *391 (3.0)* | *1 (0.0)* | *392 (2.0)* |
| *3.C* | *P02.1* | *Fetus and newborn affected by other forms of placental separation and hemorrhage. Abruptio placentae Accidental hemorrhage Antepartum hemorrhage Damage to placenta from amniocentesis, caesarean section or surgical induction Maternal blood loss* | *350 (2.6)* | *11 (0.2)* | *361 (1.9)* |
|  |  |  |  |  |  |
| 4 | R95-R99 | Sudden infant death syndrome and unknown causes of mortality | 280 (2.1) | 1,696 (27.9) | 1,976 (10.2) |
| *4.A* | *R95* | *Sudden infant death syndrome* | *159 (1.2)* | *1285 (21.2)* | *1,444 (7.5)* |
| *4.B* | *R99* | *Other ill-defined and unspecified causes of mortality Including Death Unknown cause of mortality* | *121 (0.9)* | *411 (6.8)* | *532 (2.8)* |
|  |  |  |  |  |  |
| 5 | Q20-Q28 | Congenital malformations of the circulatory system | 779 (5.9) | 718 (11.8) | 1,497 (7.8) |
| *5.A* | *Q24.9* | *Congenital malformation of heart, unspecified* | *293 (2.2)* | *261 (4.3)* | *554 (2.9)* |
| *5.B* | *Q23.4* | *Hypoplastic left heart syndrome Atresia, or marked hypoplasia of aortic orifice or valve, with hypoplasia of ascending aorta and defective development of left ventricle (with mitral valve stenosis or atresia).* | *144 (1.1)* | *107 (1.8)* | *251 (1.3)* |
| *5.C* | *Q21.3* | *Tetralogy of Fallot Ventricular septal defect with pulmonary stenosis or atresia, dextroposition of aorta and hypertrophy of right ventricle.* | *21 (0.2)* | *53 (0.9)* | *74 (0.4)* |
|  |  |  |  |  |  |
| 6 | Q90-Q99 | Chromosomal abnormalities, not elsewhere classified | 803 (6.1) | 312 (5.1) | 1,115 (5.8) |
| *6.A* | *Q91.3* | *Edwards syndrome, unspecified* | *398 (3.0)* | *166 (2.7)* | *564 (2.9)* |
| *6.B* | *Q91.7* | *Patau syndrome, unspecified* | *255 (1.9)* | *49 (0.8)* | *304 (1.6)* |
| *6.C* | *Q90.9* | *Down syndrome, unspecified Trisomy 21* | *47 (0.4)* | *33 (0.5)* | *80 (0.4)* |
|  |  |  |  |  |  |
| 7 | Q00-Q07 | Congenital malformations of the nervous system | 567 (4.3) | 186 (3.1) | 753 (3.9) |
| 8 | P50-P61 | Hemorrhagic and hematological disorders of fetus and newborn | 559 (4.2) | 21 (0.3) | 580 (3.0) |
| 9 | Q65-Q79 | Congenital malformations and deformations of the musculoskeletal system | 424 (3.2) | 122 (2.0) | 546 (2.8) |
| 10 | Q80-Q89 | Other congenital malformations | 392 (3.0) | 143 (2.4) | 535 (2.8) |
| 11 | P35-P39 | Infections specific to the perinatal period | 401 (3.0) | 35 (0.6) | 436 (2.3) |
| 12 | Q60-Q64 | Congenital malformations of the urinary system | 406 (3.1) | 30 (0.5) | 436 (2.3) |
| 13 | P75-P78 | Digestive system disorders of fetus and newborn | 309 (2.3) | 67 (1.1) | 376 (1.9) |
| 14 | W75-W84 | Other accidental threats to breathing | 48 (0.4) | 281 (4.6) | 329 (1.7) |
| 15 | Q30-Q34 | Congenital malformations of the respiratory system | 258 (2.0) | 56 (0.9) | 314 (1.6) |
| 16 | P90-P96 | Other disorders originating in the perinatal period | 231 (1.7) | 36 (0.6) | 267 (1.4) |
| 17 | X85-Y09 | Assault | 32 (0.2) | 221 (3.6) | 253 (1.3) |
| 18 | P80-P83 | Conditions involving the integument and temperature regulation of fetus and newborn | 187 (1.4) | 8 (0.1) | 195 (1.0) |
| 19 | I30-I52 | Other forms of heart disease | 53 (0.4) | 134 (2.2) | 187 (1.0) |
| 20 | E70-E90 | Metabolic disorders | 68 (0.5) | 97 (1.6) | 165 (0.9) |
| 21 | A30-A49 | Other bacterial diseases | 15 (0.1) | 138 (2.3) | 153 (0.8) |
| 22 | J09-J18 | Influenza and pneumonia | 2 (0.0) | 138 (2.3) | 140 (0.7) |
| 23 | A00-A09 | Intestinal infectious diseases | 3 (0.0) | 118 (1.9) | 121 (0.6) |
| 24 | J95-J99 | Other diseases of the respiratory system | 1 (0.0) | 98 (1.6) | 99 (0.5) |
| 25 | Q38-Q45 | Other **congenital malformations** of the digestive system | 36 (0.3) | 59 (1.0) | 95 (0.5) |
| 26 | I60-I69 | Cerebrovascular diseases | 5 (0.0) | 84 (1.4) | 89 (0.5) |
| 27 | G90-G99 | Other disorders of the nervous system | 20 (0.2) | 58 (1.0) | 78 (0.4) |
| 28 | Y10-Y34 | Event of undetermined intent | 15 (0.1) | 59 (1.0) | 74 (0.4) |
| 29 | G10-G14 | Systemicatrophies primarily affecting the central nervous system | 6 (0.0) | 64 (1.1) | 70 (0.4) |
| 30 | G00-G09 | Inflammatory diseases of the central nervous system | 19 (0.1) | 37 (0.6) | 56 (0.3) |
| 31 | G70-G73 | Diseases of myoneural junction and muscle | 11 (0.1) | 42 (0.7) | 53 (0.3) |
| 32 | B25-B34 | Other viral diseases | 6 (0.0) | 45 (0.7) | 51 (0.3) |
|  |  | Subtotal for first 32 conditions | 12,988 (98.2) | 5,371 (88.4) | 18,359 (95.1) |
|  |  | Remaining causes of mortality | 240 (1.8) | 702 (11.6) | 942 (4.9) |
|  |  | Total | 13,228 (68.5) | 6,073 (31.5) | 19,301 (100) |

^a^ International Statistical Classification of Diseases and Related Health Problems 10th Revision (ICD-10)-WHO Version for 2016 https://icd.who.int/browse10/2016/en#/VII
